# Comparative analysis of the mitochondrial genomes of the soft-shelled turtles *Palea steindachneri* and *Pelodiscus axenaria* and phylogenetic implications for Trionychia

**DOI:** 10.1038/s41598-025-90985-2

**Published:** 2025-02-28

**Authors:** Chen Chen, Liqin Ji, Guiyun Huang, Xiaoli Liu, Haigang Chen, Yakun Wang, Lingyun Yu, Yihui Liu, Xiaoyou Hong, Chengqing Wei, Congcong Wu, Laifu Luo, Xinping Zhu, Wei Li

**Affiliations:** 1 Key Laboratory of Tropical and Subtropical Fishery Resources Application and Cultivation, Ministry of Agriculture and Rural Affairs, Pearl River Fisheries Research Institute, Chinese Academy of Fishery Sciences, Guangzhou, 510380 China; 2 Department of Agriculture of Guangdong Province, Agro-Tech Extension Center of Guangdong Province, Guangzhou, China

**Keywords:** Trionychids, Control region, Selective pressure, Phylogenesis, Phylomitogenomics, Bayesian relaxed clock analysis, Mitochondrial genome, Evolutionary biology, Comparative genomics

## Abstract

Soft-shelled turtles, or Trionychia, are an enigmatic and fascinating group due to their specific morphological features and ecological adaptations. Based on mitochondrial DNA (mtDNA) and/or nuclear markers, previous studies showed the incongruent phylogenetic topologies within Trionychia (e.g., the *Palea* and its closely related species). In order to resolve the equivocal relationships and obtain some “genome-level” common evolutionary characters of soft-shelled turtles, in this study, we assembled and annotated the complete mitochondrial genomes of *Palea steindachneri* and *Pelodiscus axenaria*, both naturally distributed in Asia. The sizes of the two mitochondrial genomes were 16,811 bp and 17,143 bp, respectively. Typical vertebrate animal mtDNA features were observed, such as the usual gene components and arrangements (37 genes with a non-coding control region) and the A + T biased nucleotide compositions on the light strand (61.5% and 62.7%, respectively). All conserved blocks common to the vertebrates control region except for the extended terminal associated sequences (ETAS2) were found in the two soft-shelled turtles. The ω ratio averaged over all sites of each protein-coding gene (PCG) was below 1, which indicated purifying selection at the gene-wide level. However, a positive selection site at the 350-codon position in the *cytb* gene was detected, as estimated by Bayes empirical Bayes (BEB) analysis. Compared with the gene subsets, the mitogenomes provided the most robust phylogenetic resolution. The monophyly of the clades Amydona, Gigantaesuarochelys, and Apalonia was well supported. Topology discrepancies were observed among different datasets (e.g., the positions of *Lissemys* and *Palea*), reflecting the heterogeneous phylogenetic signals in the soft-shelled turtle mitogenomes. Precise date estimation based on Bayesian relaxed clock analyses indicated that the crown group age of extant Trionychia was approximately 115.84 Ma (95% HPD: 91.33–142.18 Ma). Paleoclimate changes, especially the Eocene − Oligocene transition, could be responsible for the speciation in these groups. Our results reiterated the necessity and effectiveness of incorporating entire mitochondrial genomes to delineate phylogenetic relationships in chelonian phylogeny studies.

**Supplementary Information:**

The online version contains supplementary material available at 10.1038/s41598-025-90985-2.

## Introduction

Soft-shelled turtles, or Trionychia, constitute an ancient and enigmatic group with a widespread geographic distribution. These turtles originated in Asia during the early Cretaceous and subsequently globally dispersed during two periods of global warming^1^. The extant soft-shelled turtles encompass 35 living species distributed across two families, Carettochelyidae and Trionychidae^2^.

Trionychia are renowned for their highly evolved morphological features, including a flattened shell, a reduced bony disc relative to the total carapace, and the complete loss of keratinized epidermal scutes, which allow them to fully adapt to aquatic environments^3–5^. The latent phylogenetic signals associated with their morphology provide valuable insights specifically into the evolutionary tempo and mode of diversification of soft-shelled turtles in comparison to other chelonian groups. In a pioneering analysis, Meylan^3^ grouped the 22 extant trionychid species into 6 tribes according to 113 osteologic characters.

However, the results of morphological studies have been challenged by findings from molecular research. Engstrom et al.^4^ reanalyzed Meylan’s morphological data and constructed phylogenetic trees via a combined dataset that included *cytb*, *nad*4 (comprising 23 bp of *tRNA*^His^), and the nuclear R35 intron. Their results revealed that most of the previously recognized tribes were not monophyletic. Using statistical methods (maximum likelihood and Bayesian analysis), Engstrom presented a consensus phylogenetic tree. These findings provided the basis for a series of further studies^2,6–10^.

On the other hand, alternative topologies are also emerging, such as a pectinate structure involving *Palea*, *Lissemys*, *Apalone*, *Dogania*, and *Pelodiscus*^11^ or (*Pelodiscus*, (*Dogania*, (*Nilssonia*, (*Amyda*, *Palea*))^12^. More recently, both Li et al.^13^ and Zhang et al.^14^ proposed a relationship that weakly positioned *Rafetus* as a sister group to the so-called Asian turtle clade composed of *Pelodiscus*, *Palea*, *Dogania*, *Amyda*, and *Nilssonia*.

These previous studies collectively provide robust support for the monophyly of Trionychia. However, ambiguities remain in the phylogenetic position of some specific tip nodes. Therefore, incorporating new sequences or taxa to increase the resolution of phylogenetic inference is desirable^15–18^.

Wattle-necked soft-shelled turtles (*Palea steindachneri*, Siebenrock 1906) and sand soft-shelled turtles (*Pelodiscus axenaria*, Zhou Zhang & Fang 1991) both belong to Trionychinae. *P. steindachneri* is the only valid species within the monotypic genus *Palea*. It is a kind of large soft-shelled turtle, with an adult weight and carapace length reaching 45 kg and 450 mm, respectively^19,20^. *P. steindachneri* is naturally distributed in Laos, Vietnam, and Southwest China, including the islands of Hong Kong and Hainan; it has also been documented in Hawaii and Mauritius as introduced species^21^. Unfortunately, over the past two decades, wild *P. steindachneri* populations have experienced a substantial decline, which is attributed primarily to anthropogenic hunting and habitat degradation. It has been defined as ‘Critically Endangered’ by the IUCN Red List and is listed in CITES Supplementary Appendix II.

In contrast, *P. axenaria* is a smaller soft-shelled turtle, with adults typically weighing no more than 500 g. According to genetically verified records, *P. axenaria* is restricted in distribution to Hunan, Guangxi, and Guangdong provinces. *P. axenaria* was initially described by Zhou et al. as ‘*axenaria*’, a term that may imply a sandy habitat, indicating its ecological niche. Recently, Gong et al.^22^ designated a neotype because of the absence of a holotype in the original description. Although *P. axenaria* is not included on the IUCN Red List, the survival status of this species is concerning.

Mitochondrial genomes, also referred to as mitogenomes, represent the sole extranuclear genome in the animal cytoplasm. They play pivotal roles in oxygen usage and energy metabolism. Vertebrate mitochondrial genomes are well known as circular, typically spanning14–20 kilobase pairs (kbp), and contain genes for 13 proteins, 2 ribosomal RNAs, 22 transfer RNAs, and 2 non-coding regions. Considered to contain useful phylogenetic information, mitochondrial genomes have been extensively studied for biological and evolutionary analyses for conservation purposes across various taxonomic levels^10,23–28^. Moreover, relative to gene fragments, mitochondrial genomes inherently have more comprehensive informational content and are thus instrumental in enhancing the accuracy and statistical robustness of internal node recovery within phylogenetic analyses^29,30^. On the other hand, exploring the evolutionary process can benefit from utilizing the entire mitogenome by accounting for the variation in evolutionary rates across the whole mitochondrial genomic range, which provides the most precise date estimates.

In the present study, the complete mitogenomes of *P. steindachneri* and *P. axenaria* were sequenced and compared with those of other Trionychia species available in GenBank. Our objectives were as follows: (1) to identify a set of “genome-level” characteristics of the two soft-shelled turtles, such as the mitochondrial nucleotide composition and structural features; (2) to determine a robust phylogenetic framework that clarifies the equivocal relationships within Trionychia; (3) to estimate the timing and patterns of soft-shelled turtle evolution using the Bayesian relaxed clock method based on mitochondrial genomes.

## Materials and methods

### Sample collection and DNA extraction

The animal collection and handling and tissue sampling procedures were approved by the Laboratory Animal Ethics Committee of the Pearl River Fisheries Research Institute, CAFS (LAEC-PRFRI-2021-05-01). All experimental methods were performed in accordance with the relevant guidelines and regulations, including the ARRIVE guidelines and the institutional animal care and use guidelines. The *P. steindachneri* sample was collected from a turtle breeding farm in Guiping City (23° 38’ N, 113° 07’ E), Guangxi Province, China, in November 2017. The *P. axenaria* individual was a wild-caught female obtained from Yongzhou City (26° 34’ N, 111° 50’ E), Hunan Province, China, in October 2020. To minimize harm to the animals, a small amount of calipash tissue above the right hind limb was clipped from each individual after anesthesia with MS-222. The tissue samples were immediately stored in 95% ethanol solution and deposited in our laboratory specimen bank. Upon completion of the sampling, the turtles emerged from anesthesia and were released back to the same sites.

Total genomic DNA was extracted from each sample via a MicroElute Genomic DNA Kit (Omega Biotek, USA) following the manufacturer’s recommendations. After concentration and integrity assessment via 1% agarose gel electrophoresis, all the DNA samples were stored at − 20 °C.

### DNA sequencing and assembly

#### P. steindachneri

The mitochondrial genome sequences of *P. steindachneri* were acquired using a series of eleven pairs of consensus mitochondrial primers for Trionychidae (Supplementary Table S1), which were designed to target the conserved regions of the Trionychidae mitogenome. PCR amplification was performed as follows: initial denaturation at 94 °C for 3 min, followed by 35 cycles of denaturation at 94 °C for 60 s, annealing at 50 °C for 2 min and 30 s and elongation at 72 °C for 5 min; and an extension step of 10 min at 72 °C. The amplified PCR products were separated on a 1.2% agarose gel, purified via the HiPure Gel Pure DNA Mini Kit (Magan, China), cloned via the pMD19-T vector (Takara, Japan), and subjected to double-strand sequencing by Guangzhou Tianyi Huiyuan Gene Technology Co., Ltd. (Guangzhou, China). All sequences were visually inspected for sequencing errors and then assembled via CAP3^31^ (https://doua.prabi.fr/software/cap3).

#### P. axenaria

*P. axenaria* genomic DNA was sent to Huitong Biotechnology Co. Ltd. (Shenzhen, China), for library construction and high-throughput sequencing. The DNA library was generated via the NEB Next^®^ Ultra™ DNA Library Prep Kit for Illumina (NEB, USA). Initially, 250 ng of the genomic DNA sample was sonicated to obtain 350 bp fragments. Fragments larger than 250 bp were selected via 1.8X AMPure XP beads (Beckman Coulter) and quantified via a Qubit^®^ 3.0 fluorometer (Invitrogen, USA). The samples subsequently underwent a series of processes, including end polishing, A-tailing, and ligation with full-length Illumina sequencing adapters. Next, the genomic library was amplified on a Phusion^®^ High-Fidelity PCR Kit (Thermo Fisher Scientific, USA) according to the protocol described by Peterson et al.^32^. PCR-amplified products were pooled and purified with a DCC-5 kit, followed by a final 1.8X AMPure XP size-selection (> 250 bp) to remove primer dimers. Then, the genomic library was sequenced on an Illumina NovaSeq 6000, generating 150 bp paired-end reads. A total of 69, 260, and 650 clean reads were obtained from *P. axenaria* library. The obtained high-quality fragments were aligned with Trionychia mitochondrial genomes from NCBI to remove sequence repeats and sequencing errors, and were then assembled via SPAdes v.3.5.0^33^ with the k-mers set to 21, 45, 65, 85, and 105. Sequencing depth and coverage map of *P. axenaria* mitochondrial genome was shown in Supplementary Fig. S1.

### Mitochondrial genome annotation and analysis

The mitochondrial genome was annotated via MITOS Web Server (http://mitos.bioinf.uni-leipzig.de/index.py), NCBI ORF finder, and tRNA-scan SE^34^ (http://lowelab.ucsc.edu/tRNAscan-SE/). Following manual examination, the fully annotated mitogenomes were submitted to GenBank under accession numbers OL405264 for *P. steindachneri* and OR805132 for *P. axenaria*. Gene maps were drawn using OrganellarGenomeDRAW (OGDRAW) v1.3.1^35^ (https://chlorobox.mpimp-golm.mpg.de/OGDraw.html). The base composition and codon usage were analyzed with MEGA X^36^ software and graphically presented using an in-house R script. The AT skew = (A − T)/(A + T) and GC skew = (G − C)/(G + C) were calculated using the method reported by Perna and Kocher^37^ in 1995. To evaluate the base bias across the entirety of the mitogenomes, calculations were conducted on the full mitogenomes and each distinct gene region. Repetitions of the control region were identified with Tandem Repeats Finder^38^ (https://tandem.bu.edu/trf/trf.html). Referring to Walberg and Clayton^39^, Sibsa et al.^40^ and Dauzery and Randi^41^, the motif of the extended termination associated sequence (ETAS) domain and conserved sequence blocks within the control region were detected with the help of MAST^42^.

### Protein-coding gene substitution rates

The ratio of nonsynonymous substitutions (Ka)/synonymous substitutions (Ks), often called Ka/Ks or ω, is a common indicator used to assess the selective pressure acting on protein-coding sequences. To ascertain potential variations in the selective pressures experienced by the two soft-shelled turtles, we calculated the Ka and Ks, along with ω, for the protein-coding genes (PCGs) through pairwise comparisons with the other 16 Trionychia mitochondrial genomes using KaKs_Calculator 3.0^43^. PCGs under no selection, purifying (negative) selection, and positive selection are expected to have ω values of 1, < 1, and > 1, respectively.

Furthermore, to model the overall substitution rates and detect signals of positive selection, we used CodeML from the PAML package v4.10.7^44^. The sequences of each PCG gene were extracted to construct the corresponding data sets, and the inputted unrooted tree was obtained according to BEAST2 analysis (as mentioned later in the text). The M0 (one-ratio) model, which assumes one ω ratio for all sites and across all lineages, serves as the null hypothesis for the test. Next, four site-heterogeneous models, M1a (nearly neutral), M2a (positive selection), M7 (beta), and M8 (beta and ω), were incorporated, all of which allow ω to vary across codons. Likelihood ratio tests (LRTs) were subsequently performed between the nested site models^45^. Genes where the comparisons between models M1a–M2a or M7–M8 result in a significant improvement for ω > 1 were deemed to show significant signs of positive selection. Finally, Bayes empirical Bayes (BEB) inference, implemented in PAML, was used to calculate the posterior probabilities for the site classes. Codons with posterior probabilities greater than 0.95 for the positively selected sites are considered to be under positive selection^46^.

### Phylogenetic analyses

Phylogenetic analyses were based on 30 mitochondrial genomes containing our two newly sequenced mitogenomes, with *Mauremys reevesii* (FJ469674.1) and *Mauremys mutica* (NC_009330.1) as outgroups. The catalog numbers, GenBank accession numbers, collection sites and references of all individuals are listed in Supplementary Table S2.

Considering the heterogeneity of evolutionary rates among PCGs, rRNAs, and tRNAs, we constructed five empirical datasets: *D*_PRT_ = PCGs + rRNAs + tRNAs, *D*_PR_ = PCGs + rRNAs, *D*_PT_ = PCGs + tRNAs, *D*_RT_ = rRNAs + tRNAs, and *D*_P_ = PCGs alone to explore the optimal dataset for phylogenetic analysis. Each mitochondrial element was individually aligned via MAFFT in PhyloSuite v1.2.2^47^ under the default settings. Then, ambiguous characters were eliminated via GBlocks v0.91b^48^ and manually reviewed via MEGA X. After that, the aligned nucleotide sequences were concatenated via BioEdit v7.0.9.0^49^.

Phylogenetic trees were reconstructed on the basis of each dataset via Bayesian inference (BI) and maximum likelihood (ML) methods. The BI analyses were performed with MrBayes v3.2.6^50^. The optimal partitioning scheme and the most suitable substitution model for each partition were determined via PartitionFinder 2 v2.1.1^51^ by using the greedy algorithm coupled with the Bayesian information criterion (BIC). Two independent Markov chain Monte Carlo (MCMC) runs of 20,000,000 generations were conducted simultaneously, with samples taken every 1000 generations. The “burn-in” phase and the corresponding samples were discarded until the average standard deviation of split frequencies (ASDSF) fell below 0.01. The ML analyses were conducted in IQ-Tree v1.6.12^52^ via an ultrafast bootstrap approximation approach with 10,000 replicates. All phylogenetic topologies were visualized with Figtree v1.4.4 (http://tree.bio.ed.ac.uk/software/figtree/).

To compare the confidence set of the consensus tree topologies, the site-wise log-likelihoods for alternative phylogenetic topologies were calculated with RaxML v8.2.12^53^ in each dataset under the GTR + gamma model. The *p*-values of various statistical tests were evaluated in CONSEL v0.20 using the consel and catpv codes^54^. We present the results of the approximately unbiased test (AU), Kishino-Hasegawa test (KH), and Shimodaira-Hasegawa test (SH). A *p*-value less than 0.05 was considered to indicate that the corresponding topology should be ruled out.

### Divergence time estimation

The time-calibrated tree was inferred via BEAST2 v2.6.4^55^ under a relaxed clock model that follows a log-normal distribution, with the Yule process model as the tree prior. This allows for rate heterogeneity among lineages. To simplify the analysis, mitochondrial genomes of 17 taxa, each representing an individual lineage, were selected as inputs. The dataset was divided into 5 partitions via PartitionFinder 2, and the nucleotide substitution priors for each partition were determined by bModelTest v2.4^56^. To convert the molecular evolutionary substitution rate estimates to chronological time, we specified the time constraint for crown Trionychia as the calibration point, following Joyce et al.^57^, spanning from the Middle Jurassic (177.6 Ma) to the top of the Barremian (124.0 Ma). The age of the pan-trionychid fossil *Striatochelys baba* (middle-upper Eocene: Bartonian–Priabonian age, 39–35 Ma)^58^ was chosen as the minimum constraint for the Asian clade. All prior criteria and model parameters for our molecular dating analyses were set by the program BEAUti 2 v2.6.6 (https://beast.community/beauti). Two independent MCMC chains of 50 million generations were conducted, with trees sampled every 2500 generations. The convergence and stationarity of each parameter’s posterior distribution were confirmed by ESS values exceeding 200, as trace log files were inspected in Tracer v.1.5 (https://beast.community/tracer).

## Results

### Mitogenome features, nucleotide composition and strand asymmetry

**Fig. 1:**
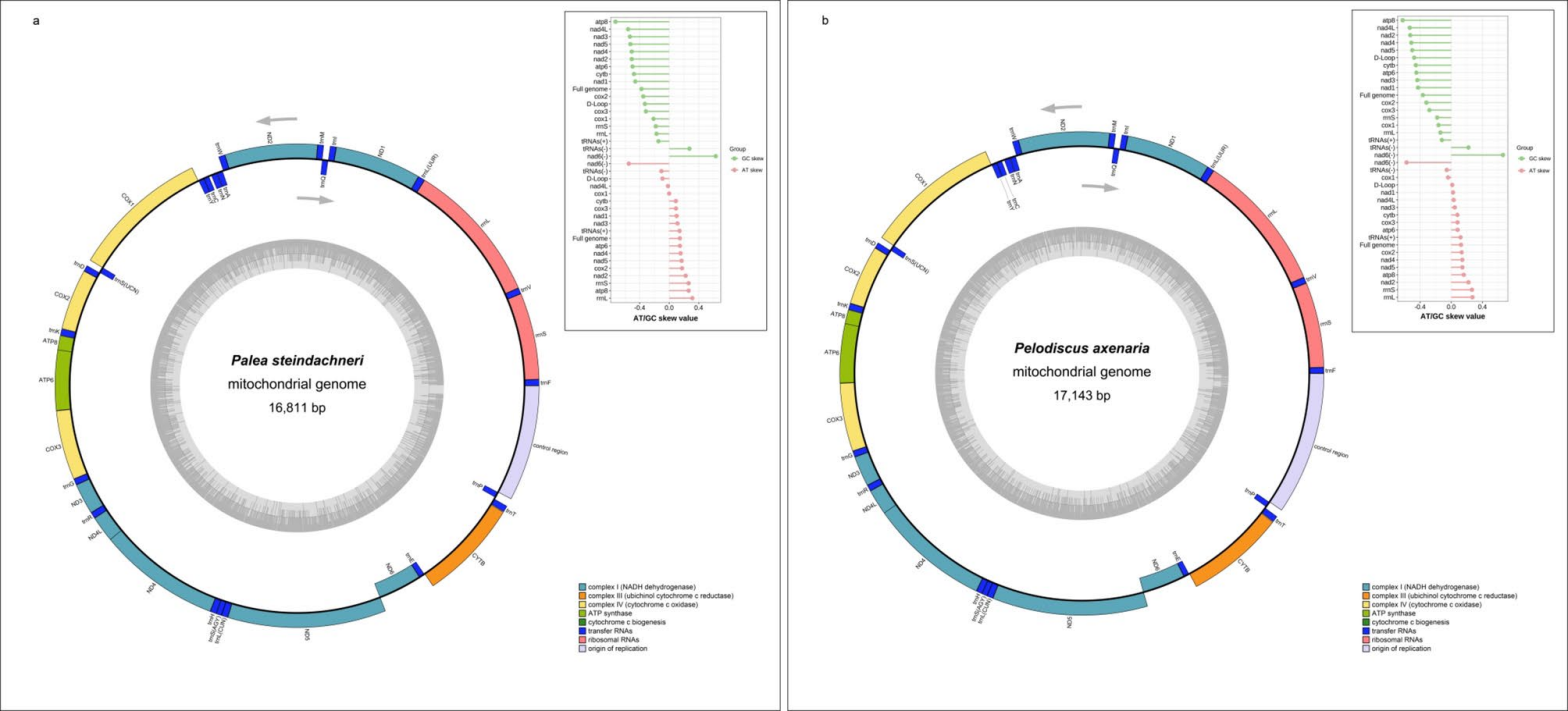
The complete mitochondrial genome maps of *Palea steindachneri* (**a**) and *Pelodiscus axenaria* (**b**). The inner circle indicates the G-C content, whereas the outer circle illustrates the mitochondrial DNA structures of the two soft-shelled turtles. The exterior and interior sections of the outer circle correspond to the L- and H-strands, respectively. The grey arrows indicate the directions of gene transcription. The subsets in the top right were AT/GC skew values of different mitochondrial elements. − indicates the L-strand, + indicates the H-strand.

The complete mitochondrial genomes of *P. steindachneri* (OL405264) and *P. axenaria* (OR805132) were 16,811 bp and 17,143 bp in size, respectively (Fig. 1). Consistent with the structure observed in other soft-shelled turtles^10^, the arrangement of genes in the two mitogenomes was characterized by the interspersion of tRNAs between the rRNAs and PCGs, and no evidence of rearrangements or duplications was detected. The nucleotide compositions of the mitochondrial genomes of both soft-shelled turtles were biased toward A and T (Table 1). In *P. steindachneri*, the A + T content was 61.5%, whereas it was 62.7% in *P. axenaria*. *tRNA*^Gly^ presented the highest A + T content, which was 72.5% in *P. steindachneri* and 72.9% in *P. axenaria*.

AT/GC skew values commonly serve as an indicator for measuring strand asymmetry. Overall, in the two species, the AT skew values for PCGs and tRNA genes on the L-strand were mostly greater than zero (except for *cox*1 and *nad*4L), whereas the GC skew values were all negative (Fig. 1). Focusing on the full L-strand, the AT/GC skew values were 0.145/−0.376 for *P. steindachneri* and 0.126/-0.364 for *P. axenaria*, and the trends of the H-strand were opposite. This finding demonstrated that both mitogenomes favor A and C strand-specific nucleotide mutation patterns on the L-strand compared with the H-strand.

**Table 1:** The mitochondrial genome features of *Palea steindachneri* (OL405264, 16,811 bp) and *Pelodiscus axenaria* (OR805132, 17,143 bp).

Mitochondrial elements	Strand	Position start/end		Size (bp)		Intergenic nucleotides (bp)		AT percentage (%)		Codon start/stop	
		*P*. st	*P*. ax	*P*. st	*P*. ax	*P*. st	*P*. ax	*P*. st	*P*. ax	*P*. st	*P*. ax
tRNA-Phe^GAA^	L	1/69	1/68	69	68	0	0	62.3	67.6	–	–
12 S-rRNA	L	70/1045	69/1045	976	977	− 2	0	58.9	59.1	–	–
tRNA-Val^UAC^	L	1044/1112	1046/1115	69	70	0	0	62.3	65.7	–	–
16 S-rRNA	L	1113/2719	1116/2714	1607	1599	0	0	61.5	63.5	–	–
tRNA-Leu^UAA^	L	2720/2795	2715/2791	76	77	0	0	52.6	53.2	–	–
*nad*1	L	2796/3767	2792/3763	972	972	− 1	− 1	59.5	62.4	ATG/TAG	ATG/TAG
tRNA-Ile^GAU^	L	3767/3836	3763/3832	70	70	− 1	− 1	57.1	57.1	–	–
tRNA-Gln^UUG^	H	3836/3906	3832/3902	71	71	− 1	− 1	69.0	71.8	–	–
tRNA-Met^CAU^	L	3906/3974	3902/3970	69	69	0	0	59.4	59.4	–	–
*nad*2	L	3975/5015	3971/5011	1041	1041	− 2	− 2	62.4	65.7	ATG/TAG	ATG/TAG
tRNA-Trp^UCA^	L	5014/5087	5010/5082	74	73	8	11	58.1	57.5	–	–
tRNA-Ala^UGC^	H	5096/5164	5094/5162	69	69	1	1	65.2	65.2	–	–
tRNA-Asn^GUU^	H	5166/5239	5164/5237	74	74	29	31	55.4	55.4	–	–
tRNA-Cys^GCA^	H	5269/5334	5269/5333	66	65	0	0	65.2	60.0	–	–
tRNA-Tyr^GUA^	H	5335/5401	5334/5399	67	66	1	1	50.7	51.5	–	–
*cox*1	L	5403/6947	5401/6945	1545	1545	− 5	− 5	58.7	60.7	GTG/AGA	GTG/AGA
tRNA-Ser^UGA^	H	6943/7013	6941/7011	71	71	0	1	60.6	60.6	–	–
tRNA-Asp^GUC^	L	7014/7082	7013/7081	69	69	0	0	71.0	69.6	–	–
*cox*2	L	7083/7769	7082/7768	687	687	1	0	62.7	63.2	ATG/TAA	ATG/TAA
tRNA-Lys^UUU^	L	7771/7843	7769/7841	73	73	1	1	57.5	54.8	–	–
*atp*8	L	7845/8009	7843/8007	165	165	− 10	− 10	64.2	67.9	ATG/TAA	ATG/TAA
*atp*6	L	8000/8683	7998/8681	684	684	− 1	− 1	61.5	65.6	ATG/TAA	ATG/TAA
*cox*3	L	8683/9466	8681/9464	784	784	0	0	56.8	58.5	ATG/T--	ATG/T--
tRNA-Gly^UCC^	L	9467/9535	9465/9534	69	70	0	0	72.5	72.9	–	–
*nad*3	L	9536/9885	9535/9884	350	350	0	0	60.7	62.5	ATG/T--	ATG/T--
tRNA-Arg^UCG^	L	9886/9955	9885/9955	70	71	0	0	67.1	77.5	–	–
*nad*4L	L	9956/10,252	9956/10,252	297	297	− 7	− 7	65.0	64.0	ATG/TAA	ATG/TAA
*nad*4	L	10,246/11,626	10,246/11,626	1381	1381	0	0	62.5	64.6	ATG/T--	ATG/T--
tRNA-His^GUG^	L	11,627/11,696	11,627/11,696	70	70	0	0	67.1	72.9	–	–
tRNA-Ser^GCU^	L	11,697/11,758	11,697/11,758	62	62	− 1	− 1	66.1	66.1	–	–
tRNA-Leu^UAG^	L	11,758/11,830	11,758/11,829	73	72	0	0	64.4	59.7	–	–
*nad*5	L	11,831/13,609	11,830/13,608	1779	1779	− 5	− 5	60.6	61.1	ATG/TAA	ATG/TAA
*nad*6	H	13,605/14,129	13,604/14,128	525	525	0	0	61.7	61.5	ATG/AGA	ATG/AGA
tRNA-Glu^UUC^	H	14,130/14,197	14,129/14,196	68	68	3	3	61.8	66.2	–	–
*cytb*	L	14,201/15,340	14,200/15,339	1140	1140	3	3	60.0	61.2	ATG/TAA	ATG/TAA
tRNA-Thr^UGU^	L	15,344/15,417	15,343/15,416	74	74	19	14	59.5	62.2	–	–
tRNA-Pro^UGG^	H	15,437/15,506	15,431/15,501	70	71	0	0	67.1	62.0	–	–
Control region	L	15,507/16,811	15,502/17,143	1305	1642	0	0	69.8	64.3	–	–

The superscripts of tRNA represent the anticodons; ‘Strand’ refers to the coding strand of each mitochondrial element; negative numbers for intergenic nucleotides indicate overlaps; T– indicates an incomplete stop codon that is completed by the addition of 3’ A residues to the mRNA.

### Protein‑coding genes and codon usage

**Fig. 2:**
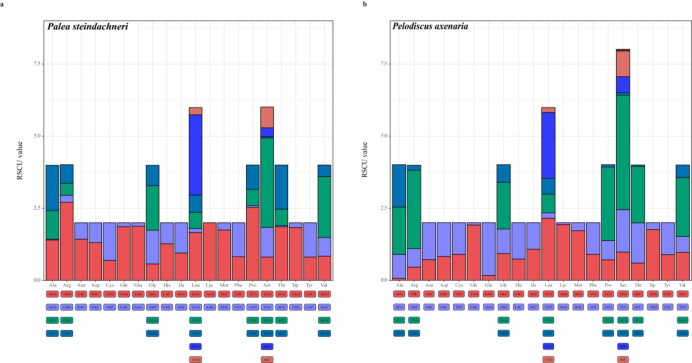
Relative synonymous codon usage (RSCU) of the mitochondrial genomes of *Palea steindachneri* (**a**) and *Pelodiscus axenaria* (**b**). The codon families of each protein-coding gene (PCG) are below the X-axis, and the stop codon is not given.

A total of 3772 amino acids were encoded in both species. All of the codons utilized in the mitochondrial genomes of *P. steindachneri* and *P. axenaria* had comparable frequencies (Supplementary Table S3). In *P. steindachneri*, the most frequently employed amino acid was leucine (CUA), followed by methionine (AUA) and threonine (ACA). Similarly, in the *P. axenaria*, the most frequently used amino acid was also Leucine (UUA), followed by Methionine (AUA) and Threonine (ACA). Across both species, amino acids encoded by A + T-rich codons (Phe, Ile, Met, Tyr, Asn, and Lys) were more frequent than those encoded by G + C-rich codons (Pro, Ala, Arg, and Gly). The ratio of A + T-rich codons to G + C-rich codons was 1.60 in *P. steindachneri*, whereas it was 1.56 in *P. axenaria*. Relative synonymous codon usage (RSCU) values greater than one were observed predominantly for A-ending or C-ending codons, indicating that the mutation bias favored A and C at the neutral (or nearly neutral) third codon position of the two soft-shelled turtle mitochondrial genomes (Fig. 2).

### Transfer and ribosomal RNAs

Consistent with the typical pattern observed in vertebrate animals, the complete set of 22 tRNA genes included two tRNAs each for serine and leucine, and one tRNA for each of the other 18 amino acids. All tRNAs, apart from *tRNA*^Ser (GCU)^, can fold into canonical clover-leaf secondary structures. The dihydrouracil (DHU) arm of *tRNA*^Ser (GCU)^ was absent, resulting in a simplified loop structure (Supplementary Fig. S2). Nevertheless, the absence of a stable DHU-arm in *tRNA*^Ser (GCU)^ seems to be a common feature in nearly all metazoan species^59^.

The two rRNA genes were both located on the L-strand between *tRNA*^Phe^ and *tRNA*^Val^. The A + T content was greater than the G + C content, accounting for 58.9% (*P. steindachneri*) and 59.1% (*P. axenaria*) of the 12 S rRNA and 61.5% (*P. steindachneri*) and 63.5% (*P. axenaria*) of the 16 S rRNA (Table 1). Consistently, the AT-skew of rRNA genes was slightly positive, whereas the GC-skew was strongly negative (Fig. 1).

### Control region

The control region is bound by the *tRNA*^Pro^ and *tRNA*^Phe^ genes in both soft-shelled turtles, with lengths of 1305 bp in *P. steindachneri* and 1642 bp in *P. axenaria*. The region presented greater size variation, which contributed to the difference in the length of the whole mitogenome. Several tandem repeat units (TRs) were identified in control regions. In *P. steindachneri*, two TRs were found. The first TR consists of five copies of a 51 bp motif, ranging from nucleotide positions 15507 to 15760, with the sequence 5’-ATTTTAC(-/T)TTTTTTTTCTCTCCCGCGCCCAAGAGATATAAAACCCCTGTAT-3’. The second TR consists of 33 copies of a 5 bp motif, ranging from nucleotide positions 16,645 to 16,809, with the sequence 5’-ATATT-3’. In *P. axenaria*, four TRs were identified. The largest motif spans 50 bp, ranging from nucleotide positions 15,503 to 15,802, with the sequence 5’-TTACGCTTTTTTTCTTCTCCCGC(A/G)CCCAAGAGACATTCTACCCCTATATT-3’.

**Fig. 3:**
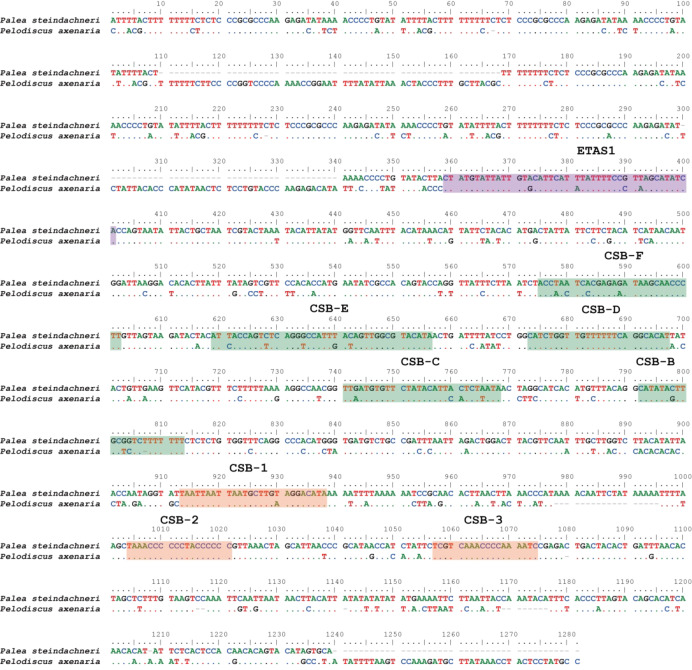
Alignment of the control region (L-strand) of *Palea steindachneri* and *Pelodiscus axenaria*. Dots indicate nucleotides identical between the two soft-shelled turtles; dashes indicate indels. The mammalian ETAS1-like sequence at nt 359–401 is colored in purple. Sequences with similarity to conserved blocks B, C, D, E, and F are colored in green. Sequences with similarity to conserved blocks 1, 2, and 3 are colored in orange.

In addition, conserved sequence regions identified in previous studies on mammalian mitochondrial DNA, including the extended termination-associated sequence (ETAS1) at the 3’ end of H-stand, central conserved domain (CSB-B, CSB-C, CSB-D, CSB-E, and CSB-F), and conserved sequence blocks (CSB-1, CSB-2, and CSB-3) at the 5’ ends of H-strand, were also reported in the two soft-shelled turtles (Fig. 3).

### Signatures of nonneutral evolution in PCGs

**Fig. 4:**
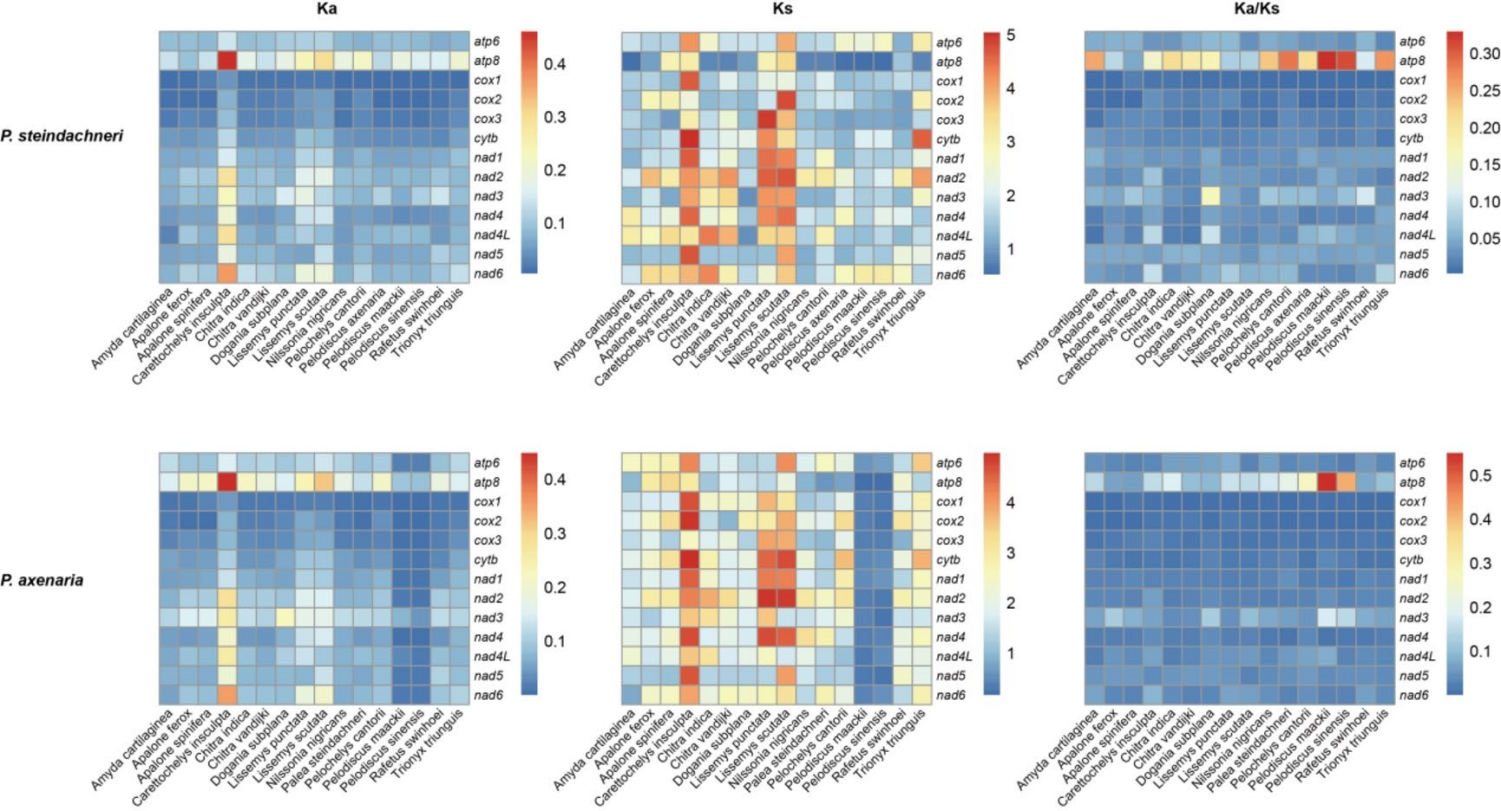
Pairwise Ka, Ks, and ω values for the 13 mitochondrial PCGs of *Palea steindachneri* and *Pelodiscus axenaria* compared with those of the other 16 Trionychia. Ka, nonsynonymous substitution rate; Ks, synonymous substitution rate; ω, Ka/Ks ratio.

The ω values of the 13 PCGs were all less than 1 in both soft-shelled turtles, indicating that these PCGs generally evolved under purifying selection (Fig. 4). However, the *atp*8 gene presented the greatest number of nonsynonymous changes and a higher ω value among the mitochondrial protein-coding genes, which suggests that selective pressures may tend to be relaxed on these genes. Additionally, the genes encoding the NADH dehydrogenase complex (*nad*1-6, *nad*4L) presented a greater number of amino acid changes than did the genes encoding cytochrome c oxidase subunits (*cox*1-*cox*3) and cytochrome b (*cytb*), with an overall greater frequency of nonsynonymous substitutions. Site model comparisons indicated that the LRT values of 4 PCGs’ (*cox*2, *cox*3, *cytb*, and *nad*5) for M7 vs. M8 were highly significant at the 5% level (Supplementary Table S4). According to the BEB analysis, 11 codons had a probability > 50% for positive selection with ω > 1, although there was significance (PP > 0.95) for only one codon in *cytb* (350 T) (Table 2, Supplementary Fig. S3).

**Table 2:** PCG identification of putative positively selected codons according to site models performed in PAML.

PCG	Model code	ℓ	Estimate parameter	Positively selected codon	posterior mean ± SE for ω	PP (ω > 1)
*cytb*	M0 (one-ration)	− 6715.6	ω = 0.0539	43 A	1.197 ± 0.473	0.596
	M1a (nearly neutral)	− 6561.86	*p*_0_ = 0.9046, *p*_1_ = 0.0954 ω_0_ = 0.0262	350 T	1.919 ± 1.056	0.988*
	M2a (positive selection)	− 6561.86	*p*_0_ = 0.9046, *p*_1_ = 0.0744, *p*_2_ = 0.0211 ω_0_ = 0.0262	361 T	1.157 ± 0.587	0.553
	M7 (beta)	− 6505.59	*p* = 0.1651, *q* = 1.5616			
	M8 (beta & ω)	− 6496.15	*p*_0_ = 0.9922, *p*_1_ = 0.0078, *p* = 0.1705 *q* = 1.9541 ω = 1.3587			

*Indicates codons identified as positively selected with posterior probability > 0.95 estimated by Bayes empirical Bayes (BEB) analysis. The parameters are shown for only PCGs in which positive selection codons were identified. All 13 PCG results are listed in Supplementary Table S4. The numbers in the positively selected codon column indicate the position of the codon, and the capital letter represents the codon abbreviation. Other abbreviations: *ℓ*, log likelihood score; PP, posterior probability.

### Phylogenetic analyses

**Fig. 5:**
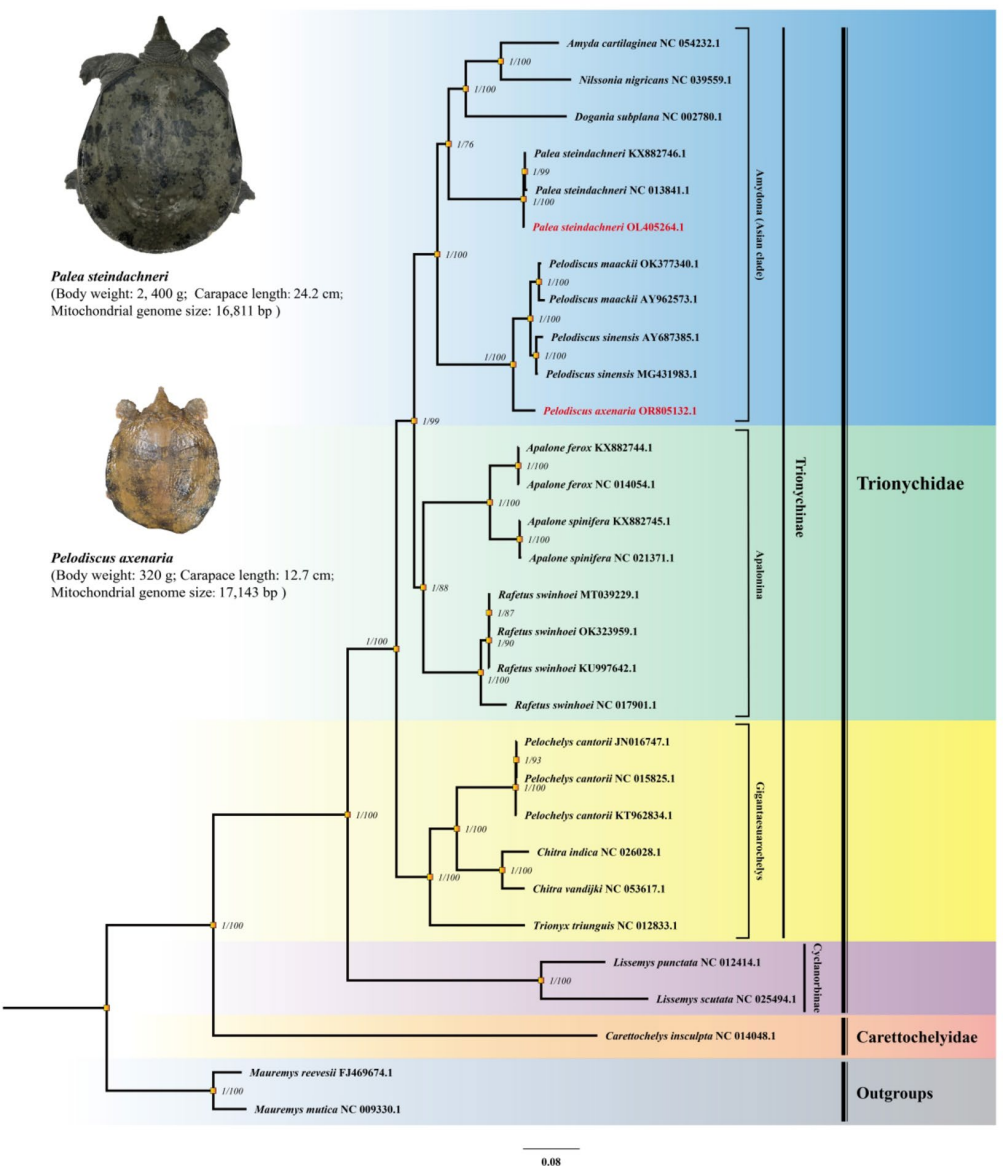
Phylogenetic analyses of Trionychia species based on the *D*_PRT_ dataset. The numbers next to the nodes represent the posterior probabilities from the Bayesian inference (BI) and the bootstrap support values for the maximum likelihood (ML) inference. *Palea steindachneri* and *Pelodiscus axenaria*, newly obtained in this study, are marked in red font. *Mauremys reevesii* and *Mauremys mutica* are outgroups. The sequence partition scheme and nucleotide substitution models for Bayesian analysis and maximum likelihood are shown in Supplementary Table S6.

Dataset *D*_PRT_ with five partitions was demonstrated to be the best scheme according to the CONSEL test results and the internal nodal support (Supplementary Table S5, Supplementary Fig. S4). Both the ML and BI analyses revealed identical topologies (Fig. 5). In the BI analysis, all posterior probabilities (PPs) reached the maximum value of 1, indicating strong support across the fully resolved phylogeny. However, in the ML analysis, some clades had moderate bootstrap support (BP) values (e.g., BP = 76% for the node of *Palea* and *Dogania* + *Nilssonia* + *Amyda*).

On the basis of the optimal topology, the monophyly of Trionychidae and Carettochelyidae was well supported (PP = 1, BP = 100%). There are four monophyletic clades within Trionychidae. The Cyclanorbinae clade, which is represented by *Lissemys*, diverged early and was identified as the sister clade to the other Trionychidae. This clade exhibited the longest branch length, which reflects a greater number of nucleotide substitutions compared to the other soft-shelled turtles. The Gigantaesuarochelys clade, consisting of giant or large soft-shelled turtles—*Pelochelys*, *Chitra*, and *Trionyx*—consistently appeared as a monophyletic group with clear support across all datasets and partition schemes (Fig. 5, Supplementary Fig. S4).

The Apalonia clade comprises *Rafetus* and *Apalone*. This phylogenetic topology was consistent with Engstrom’s best estimations^4^ and Le’s findings^60^, which were based on combined mitochondrial and nuclear DNA sequence data. In line with these two studies, the sister relationship between *Rafetus* and *Apalone* was relatively weak in the ML analysis (BP = 88%) in our results.

*P. steindachneri* and *P. axenaria* both clustered within the Amydona clade (Asian clade). *P. axenaria* was closely related to *P. sinensis* and *P. maackii*, which were resolved as reciprocally monophyletic groups. *P. steindachneri* is located at the base position of its successive sister groups, *Dogania*, *Nilssonia*, and *Amyda*. The nodal support of this grouping was 76% in the ML bootstrap with 1.00 in the Bayesian posterior.

### Divergence time estimation

**Fig. 6:**
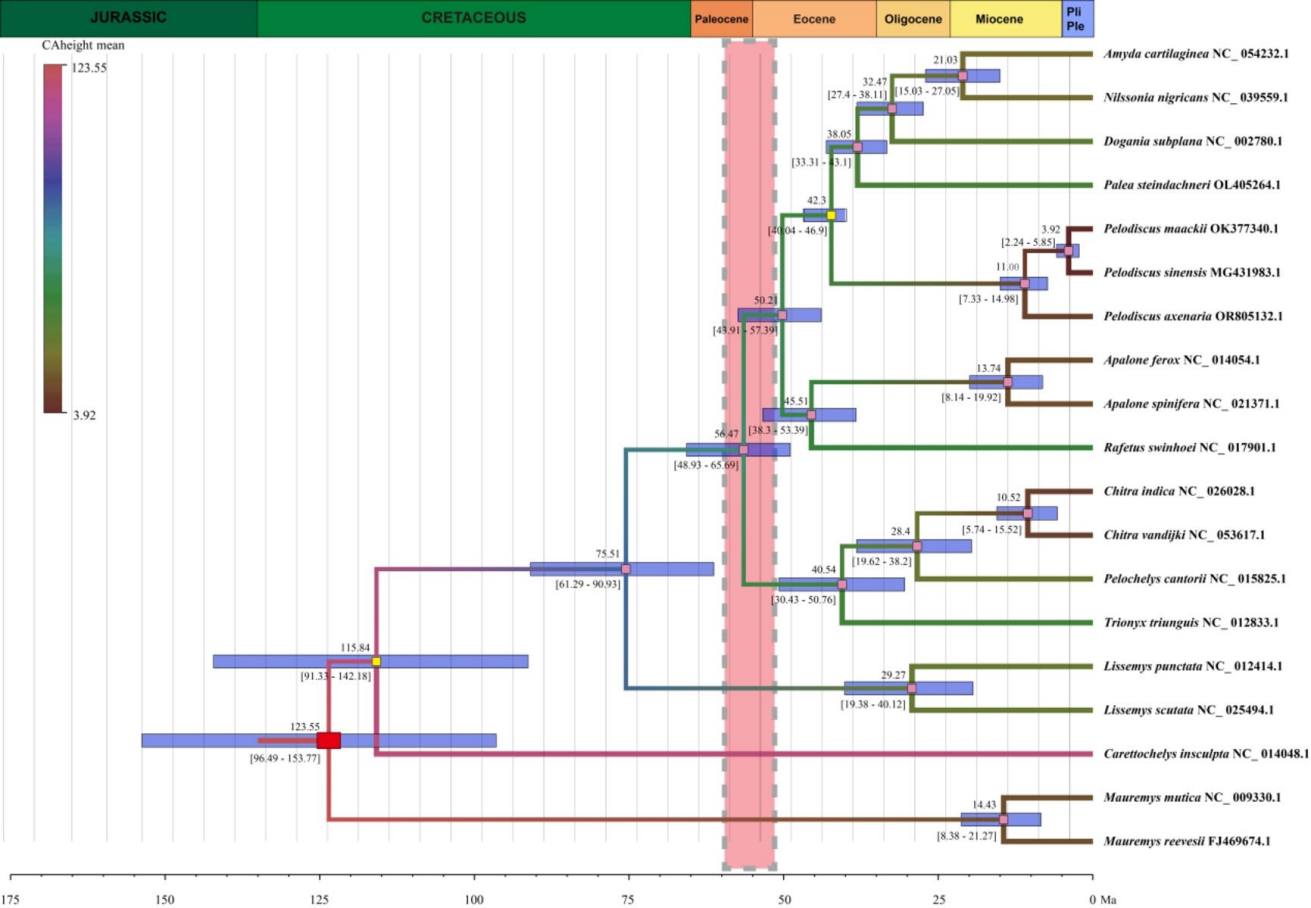
The chronogram of Trionychia inferred from Bayesian relaxed clock analyses based on the mitochondrial genomes. The red rectangular block represents the root node, two yellow square blocks represent the calibration points, and light blue horizontal bars indicate the 95% HPD range of the node heights. Numbers above the bars are the estimated mean ages, while numbers below indicate the 95% HPD values. The pink shaded area represents the first global warming period. Priors for divergence time estimation are shown in Supplementary Table S7.

The chronogram based on the Bayesian relaxed clock analyses of soft-shelled turtle genomes is shown in Fig. 6. Stem age of Trionychia was inferred to have originated in the Cretaceous, ~ 123.55 Ma (95% HPD: 96.49–153.77 Ma), while the estimated crown group age of living Trionychia was ~ 115.84 Ma (95% HPD: 91.33–142.18 Ma). Within Trionychidae, the estimated age of the divergence between Cyclanorbinae and Trionychinae was ~ 75.51 Ma with an interval of 61.29–90.93 Ma. Interestingly, we found that the most recent common ancestor (MRCA) of Trionychinae emerged around the first global warming period (~ 58–52 Ma). Major split occurred within 10 million years after the early Eocene Climatic Optimum (52–50 Ma), rapidly forming the extant three clades.

## Discussion

In this research, we determined the complete mitochondrial genomes of *P. steindachneri* and *P. axenaria*. We compared the mitogenome features, codon usage, and Ka/Ks ratios of the 13 protein-coding genes and analyzed the characteristics of the control region. Additionally, molecular clock and phylogenetic frameworks were also tested using the currently available mitochondrial genome data from 17 taxa belonging to 2 families of Trionychia. Our results suggested several important ideas about the general characteristics of soft-shelled turtle mitogenomes and potentially explained some of the contradictory phylogenetic relationships inferred from previous studies.

### Patterns of nucleotide composition and strand asymmetry

The A + T content, AT-skew, and GC-skew are three key parameters commonly employed to explore the patterns of nucleotide composition. Our findings revealed a bias in the nucleotide compositions towards A + T, along with an A and C nucleotide mutation bias on the L-strands. These patterns were also found among other vertebrate mitochondrial genomes (e.g., in mammals, see related discussion in Min and Hickey 2007^61^). The duration of the single-stranded state of the heavy strand during replication is responsible for base rates in divergent directions on the two strands, which is believed to influence the observed strand bias in animal mitochondrial genomes^61,62^. Interestingly, our results indicated that the A and C directionality of the compositional asymmetries was consistent with the preference for A-ending or C-ending codons for both mitochondrial genomes. This tendency is consistent with the hypothesis that strand-specific mutation bias is the main force driving and maintaining codon usage bias^63,64^.

### Control region structure

The mitochondrial genome structures of *P. steindachneri* and *P. axenaria* are relatively conserved overall, and the main difference was in the length of the control regions. This difference is mainly attributable to the difference in the copy number of tandem repeat units between the two species. Our results revealed that the long tandem repeat units in both soft-shelled turtles were located at the 5’ end of the control region (based on the L-strand sequence). This phenomenon confirms the hypothesis that the origins of replication, or the sites of termination of the H-strands, are prone to the insertion and deletion of elements due to replication slippage^41,65^. This variability in these tandem repeats among different individuals can be used as a tool for population genetic analysis^66^.

The ETAS domain, which contains the motif GYRCAT (Y = C or T; R = A or G), corresponds to the 3’ end of the D-loop. It has been reported to be associated with multiple bidirectional signals, such as active processing/termination of both light (L-) and heavy (H-) transcripts, as well as the termination of newly synthesized H-strands^40,67^. Two ETAS blocks, named ETAS1 and ETAS2, coexist in most mammalian and avian control regions (see Sbisa et al.^40^ 1997 and Randi and Lucchini 1998^68^). However, our results revealed that the two soft-shelled turtles possessed ETAS1, whereas ETAS2 was absent. This finding might indicate that this element is less conserved in soft-shelled turtles. The absence of ETAS2 has also been observed in Cypriniformes^69^, *Gyps himalayensis*^70^, *Accipiter virgatus*^71^, and *Anomalurus* sp.^72^.

CSB-1 to CSB-3 are located upstream of the promoters of the light (L-) and heavy (H-) strands in vertebrates. However, the occurrence of CSBs varies among different vertebrates^73^. CSB-1 is known to be present in all vertebrates. It is easily recognized by its 3’-terminal GGACATA motif^41,68^. Sbisa et al.^40^ reported that CSB-1 could be critical in primer generation for the synthesis of new H-strands, and that the complete deletion of CSB1 would reduce the mitochondrial RNA processing efficiency of RNAse. CSB2 and CSB3, both rich in C residues, displayed a degree of variation across different taxa. The two elements have been reported to be partially absent in rodents^74^, cetaceans^75^, and dolphins^76^, or possessed a single fused 2 + 3 in avian species^68^, sheep^77^, and cervids^41^. Nevertheless, according to our results, the two soft-shelled turtles contained all three CSB blocks, indicating the conserved sequence features of the control region in Trionychia.

### Protein-coding gene substitution rates

The Ka and Ks substitution rates provide insights into the evolutionary dynamics of protein-coding genes among closely related species. Our results revealed that the ω ratio of each gene, averaged over all sites, experienced purifying selection on protein-coding sequences (ω < 1) in the two soft-shelled turtle mitochondrial genomes. Lower ω values suggested stronger purifying selection acting on the nonsynonymous mutations. This pattern was also found in other vertebrate mitochondrial genomes^78–80^. Castellana et al.^81^ pointed out that the role of purifying selection is necessary for maintaining mitochondrial gene functions, since mitochondria are the site for the citric acid cycle and oxidative phosphorylation (OXPHOS). However, we also found a 350-codon position under positive selection in *cytb*, with an ω estimate of 1.919 ± 1.056 (Table 2, Supplementary Fig. S3). Although the true biological function of the nonsynonymous mutation cannot be fully interpreted here, our results reflect that, in the overall context of purifying selection, there can still be weak and/or episodic positive selection occurring.

Meanwhile, our results revealed that the *atp*8 gene presented the greatest number of nonsynonymous changes and a relatively high ω value, indicating varying levels of functional constraint among different genes involved in OXPHOS. Sun et al.^79^ concluded that energetic functional constraints represent the major factor shaping mitochondrial DNA evolution. Fewer constraints on mitochondrial proteins may allow for higher levels of nonsynonymous substitutions (dN) in mitochondrial genes. The primary role of the ATP8 subunit is limited to the assembly of the proton channel (F0 component) in the mitochondria^82^, which might not be a critical component of the respiratory chain. Therefore, in most metazoan mitochondrial genomes, this gene is consistently the most variable^62,80,82^ and even absent in most marine bivalves^83^.

### Phylogenetic relationships and divergence times

The trees based on *D*_PRT_ generally agreed with Engstrom et al.’s previous optimal topology using concatenated datasets containing two partial mitochondrial PCGs and a nuclear introns^4^. The monophyly of the Asian turtle clade was well resolved and consisted of *P. axenaria*, *P. sinensis*, *P. maackii*, *P. steindachneri*, *Dogania subplana*, *Nissonia nigricans*, and *Amyda cartilaginea* (Fig. 5).

*P. axenaria* was identified as the sister clade to *P. sinensis* + *P. maackii*. These three species are generally characterized by their small to medium body size, and the differences in their morphological features are subtle. Hence, they were believed to be the same species for a considerable period^84^. However, Fritz et al.^85^ classified these turtles as separate species and further demonstrated significant genetic differences between *P. axenaria* and other *Pelodiscus* species via analyses of three mitochondrial gene fragments and a nuclear gene, C-mos. Additionally, according to Stuckas and Fritz^84^, the uncorrected *p* distances between *P. axenaria*, *P. maackii*, and *P. sinensis*, based on *cytb* + *tRNA*^Thr^, ranged from 8.32 to 8.61%. This genetic variation is consistent with the benchmarks for species separation within other chelonian genera^86–88^. Moreover, the osteological characteristic of *P. sinensis* entoplastron forms a 90° angle, whereas the angle in *P. axenaria* is greater than 90°, according to Gong’s neotype^89^. Our results, along with previous work, collectively substantiate the separation of these three soft-shelled turtles into well-defined species.

The phylogenetic placement of *Rafetus* may reflect the challenge of assigning nodes within the phylogeny of soft-shelled turtles. Weak node support or consistently conflicting topologies between different datasets have been recurrent issues across various studies. Another common topology observed was one in which *Apalone* and *Rafetus swinhoei* diverged consecutively and then formed a pectinate structure with the Asian turtles clade^13^. While the limited breadth of taxon sampling is always acknowledged as an explanation for variations in topological structures, this factor alone falls short of offering a compelling justification^15,90,91^, particularly in addressing deeply divergent nodes, such as those found in Trionychia. Whereas too few taxa can result in numerous unobserved changes in a matrix^91^, the reduction in phylogenetic inference accuracy associated with incomplete taxa is due primarily to the absence of key characters at specific positions^17,92–94^. For this reason, simulation studies typically demonstrate that initially, adding data quickly moves the simulated tree closer to the true tree, whereas subsequent additions lead to more gradual improvements^95,96^. In our study, along with previous research, the sampled taxa encompassed most of the extant turtles. Hence, incomplete taxonomic units are not the primary factor causing differences in topology. Furthermore, in model-based analysis methods, compared with the degree of data incompleteness, model selection and data partitioning schemes are more likely to lead to varying tree topologies and branch support levels^97,98^. Both the maximum likelihood method and the Bayesian method yielded consistent topologies in our analysis, indicating the robustness of these results. Therefore, we concluded that the recovered sister relationship of *Rafetus-Apalone* reflects the complex reality of the phylogeny.

Deciphering the timescale of speciation events is crucial for understanding the evolutionary process of soft-shelled turtles. Our results revealed that the MRCA of Trionychia was at ~ 115.84 Ma (91.33-142.18 HPD) (Fig. 6). There was a slight difference compared to the findings of Le et al.^60^, the estimated age of which was at 133.46 Ma (105.8–187.65), based on the concatenated dataset of *cytb*, *nad*4, and R35. However, the narrower HPD intervals suggested that the accuracy of our estimates had increased, likely as a result of the addition of more molecular data^29^.

Our results also indicated that the speciation rate in Trionychinae was accelerated after the first global warming period. Paleoclimate changes could be responsible for the divergence in these groups. We speculated that the MRCA of extant Trionychinae flourished and dispersed around the globe during the Paleocene-Eocene thermal maximum (~ 55.5 Ma). However, with the onset of the Eocene-Oligocene transition, there was a large temperature drop in growing-season surface water (∼10 °C)^99^. The suitable habitat range for soft-shelled turtles was dramatically compressed and became highly fragmented. These environmental changes pushed forward rapid speciation events. Our findings are highly consistent with the results obtained by Thomson et al. using 15 nuclear loci^100^.

### Phylogenetic signal heterogeneity in soft-shelled mitochondrial genomes

Despite some instances in which it has been challenging to clarify the deeper phylogenetic relationships within the chelonian group on the basis of mitochondrial DNA^101–103^, our study demonstrated that the mitogenomic dataset offers substantial and robust phylogenetic insights for the vast majority of trionychids that closely align with established taxonomic classifications. This is not surprising. The smaller population size for the mitochondrial genome increases the probability that the mitochondrial gene tree will match the species tree if there is no influence of random coalescence^104^.

Due to the physical linkage of mitochondrial genes within the same DNA molecule, it is often assumed that the gene trees of all mitochondrial features represent the same genealogical history^105,106^. However, our research revealed incongruent phylogenetic results among different subsets of mitochondrial data. Compared with the whole mitochondrial genome, the concatenated rRNAs + tRNAs dataset (*D*_RT_) and the PCGs dataset (*D*_P_) resulted in three different topologies (Supplementary Fig. S4). Lower nodal support was observed within the Amydona, Apalonia, and Gigantaesuarochelys clades under BI and ML analyses (PP < 0.95, BP < 70%). Furthermore, the placement of *Lissemys* in the PCGs dataset tree was possibly influenced by long-branch attraction. This indicated that neither *D*_RT_ nor *D*_P_ has sufficient power to resolve the internal relationships. The three topologies were all ruled out by the non-parametric likelihood-based tests (Supplementary Table S5).

The trees constructed by combining two different mitochondrial elements appeared to be more powerful. The concatenated PCGs + tRNAs dataset (*D*_PT_) and PCGs + rRNAs dataset (*D*_PR_) both received strong support for most clades from both BI and ML analyses. The *D*_PT_ resulted in the same topology as the whole mitochondrial genome, while the *D*_PR_ retrieved a topology of the *Palea*/*Pelodiscus* dichotomy with weak nodal support (PP = 0.89, BP = 57%).

On the basis of our findings, we can conclude that functionally distinct classes of mitochondrial feature regions harbor their own unique phylogenetic signals. Discrepancies are likely due to varying evolutionary rates and/or selection pressures^29,107^. More conserved regions may provide lower resolution (as observed in the rRNAs + tRNAs dataset), but still contain valuable phylogenetic information. Conversely, highly variable regions can produce higher resolution (as seen in the PCGs dataset), but generate trees that do not reflect the topology of the complete mitogenome^108^. Therefore, compared with single genes or gene subsets, complete mitogenomes typically offer more robust results for phylogenetic analyses. Previous studies have encountered challenges in using mitochondrial markers for phylogenetic analysis, possibly because they utilized only a limited number of elements that do not comprehensively reflect the phylogenetic content of the mitochondrial genome. This suggests the importance of utilizing larger portions or the whole mitogenome in chelonian phylogenetic studies.

## Conclusion

As important organelles, mitochondria play crucial roles in numerous biological processes, and mitochondrial DNA provides a rich source of phylogenetic inference information. In this study, we described the complete mitochondrial genomes of two soft-shelled turtles, *P. steindachneri* and *P. axenaria*. We found that the main structural features of these mitogenomes, such as gene components and nucleotide composition, were consistent with the paradigm observed in vertebrate animals. However, the absence of the conserved blocks ETAS2 and the varying number of tandem repeat units in the control region reflected the mitochondrial genome structural variations of the soft-shelled turtles. We also found that despite the heterogeneity in phylogenetic signals across different elements of the soft-shelled turtle mitogenomes, utilizing the entire mitochondrial genome or larger portions can yield robust topologies for Trionychia, which closely align with established taxonomic classifications. Moreover, based on Bayesian relaxed clock estimation, paleoclimate changes, especially the Eocene –Oligocene transition, could be responsible for the speciation in these enigmatic groups. Our study suggests that the mitochondrial genome will continue to play an important role in phylogenetic research of species similar to soft-shelled turtles in the future.

## Electronic supplementary material

Below is the link to the electronic supplementary material.

Supplementary Material 1

Supplementary Material 2

Supplementary Material 3

## Data Availability

The mitochondrial genome sequences have been deposited in GenBank with accession numbers OL405264 (https://www.ncbi.nlm.nih.gov/nuccore/OL405264) and OR805132 (https://www.ncbi.nlm.nih.gov/nuccore/OR805132). The Illumina raw reads of P. axenaria have been submitted to the NCBI Sequence Read Archive (SRA) under the project accession number PRJNA1037746 (https://www.ncbi.nlm.nih.gov/bioproject/PRJNA1037746), with sample accession number SAMN38196774 and run accession number SRR26782263. All data are publicly accessible via the provided links.

## References

[1] Joyce, W. G., Schoch, R. R. & Lyson, T. R. The girdles of the oldest fossil turtle, *Proterochersis robusta*, and the age of the turtle crown. *BMC Evol. Biol.* **13**, 266. 10.1186/1471-2148-13-266 (2013). 24314094 10.1186/1471-2148-13-266 PMC4077068

[2] Guillon, J. M., Guéry, L., Hulin, V. & Girondot, M. A large phylogeny of turtles (Testudines) using molecular data. *Contrib. Zool.* **81**, 147–158. 10.1163/18759866-08103002 (2012).

[3] Meylan, P. A. The phylogenetic relationships of soft-shelled turtles (family Trionychidae). *Bull. AMNH* **186**, 1–101 (1987).

[4] Engstrom, T. N., Shaffer, H. B. & McCord, W. P. Multiple data sets, high homoplasy, and the phylogeny of softshell turtles (Testudines: Trionychidae). *Syst. Biol.* **53**, 693–710. 10.1080/10635150490503053 (2004). 15545250 10.1080/10635150490503053

[5] Vickaryous, M. K. & Sire, J. Y. The integumentary skeleton of tetrapods: origin, evolution, and development. *J. Anat.* **214**, 441–464. 10.1111/j.1469-7580.2008.01043.x (2009). 19422424 10.1111/j.1469-7580.2008.01043.x PMC2736118

[6] Praschag, P., Hundsdörfer, A. K., Reza, A. H. M. A. & Fritz, U. Genetic evidence for wild-living *Aspideretes nigricans* and a molecular phylogeny of South Asian softshell turtles (Reptilia: Trionychidae: Aspideretes, Nilssonia). *Zool. Scr.* **36**, 301–310. 10.1111/j.1463-6409.2007.00282.x (2007).

[7] Joyce, W. G. & Lyson, T. R. A neglected lineage of North American turtles fills a major gap in the fossil record. *Palaeontology* **53**, 241–248. 10.1111/j.1475-4983.2009.00929.x (2010).

[8] Praschag, P., Stuckas, H., Päckert, M., Maran, J. & Fritz, U. Mitochondrial DNA sequences suggest a revised taxonomy of Asian flapshell turtles (*Lissemys* SMITH, 1931) and the validity of previously unrecognized taxa (Testudines: Trionychidae). *Vertebr. Zool.* **61**, 147–160. 10.3897/vz.61.e31146 (2011).

[9] Lourenço, J. M., Claude, J., Galtier, N. & Chiari, Y. Dating Cryptodiran nodes: origin and diversification of the turtle superfamily Testudinoidea. *Mol. Phylogenet. Evol.* **62**, 496–507. 10.1016/J.YMPEV.2011.10.022 (2012). 22100825 10.1016/j.ympev.2011.10.022

[10] Kundu, S. et al. Complete mitochondrial genome of black soft-shell turtle (*Nilssonia nigricans*) and comparative analysis with other Trionychidae. *Sci. Rep.* **8**, 1–11. 10.1038/s41598-018-35822-5 (2018). 30478342 10.1038/s41598-018-35822-5 PMC6255766

[11] Xiong, L., Nie, L. W., Li, X. S. & Liu, X. Comparison research and phylogenetic implications of mitochondrial control regions in four soft-shelled turtles of Trionychia (Reptilia, Testudinata). *Genes Genom.* **32**, 291–298. 10.1007/s13258-010-0015-8 (2010).

[12] Pereira, A. G., Sterli, J., Moreira, F. R. R. & Schrago, C. G. Multilocus phylogeny and statistical biogeography clarify the evolutionary history of major lineages of turtles. *Mol. Phylogenet. Evol.* **113**, 59–66. 10.1016/j.ympev.2017.05.008 (2017). 28501611 10.1016/j.ympev.2017.05.008

[13] Li, H. F. et al. Phylogenetic relationships and divergence dates of softshell turtles (Testudines: Trionychidae) inferred from complete mitochondrial genomes. *J. Evol. Biol.* **30**, 1011–1023. 10.1111/jeb.13070 (2017). 28294452 10.1111/jeb.13070

[14] Zhang, J. et al. Characterization of the complete mitochondrial genome and phylogenetic analysis of *Pelodiscus sinensis*, a mutant Chinese soft-shell turtle. *Conserv. Genet. Resour.* **11**, 279–282. 10.1007/S12686-018-1007-2 (2019).

[15] Townsend, J. P. & Lopez-Giraldez, F. Optimal selection of gene and ingroup taxon sampling for resolving phylogenetic relationships. *Syst. Biol.* **59**, 446–457. 10.1093/sysbio/syq025 (2010). 20547780 10.1093/sysbio/syq025

[16] Heath, T. A., Hedtke, S. M. & Hillis, D. M. Taxon sampling and the accuracy of phylogenetic analyses. *J. Syst. Evol.* **46**, 239–257. 10.3724/SP.J.1002.2008.08016 (2008).

[17] Wiens, J. J. Incomplete taxa, incomplete characters, and phylogenetic accuracy: is there a missing data problem? *J. Vertebr. Paleontol.* **23**, 297–310 (2003).

[18] Bravo, G. A. et al. Embracing heterogeneity: Coalescing the tree of life and the future of phylogenomics. *PeerJ* **2019**, 1–60. 10.7717/peerj.6399 (2019). 10.7717/peerj.6399 PMC6378093 30783571

[19] Marchetti, M. P. & Engstrom, T. The conservation paradox of endangered and invasive species. *Conserv. Biol.* **30**, 434–437. 10.1111/cobi.12642 (2016). 26954433 10.1111/cobi.12642

[20] Le Duc, O. et al. Farming characteristics and the ecology of *Palea steindachneri* (Trionychidae) in Vietnam. *Russ. J. Herpetol.* **27**, 97–108. 10.30906/1026-2296-2020-27-2-97-108 (2020).

[21] Dong, C. M., Engstrom, T. N. & Thomson, R. C. Origins of softshell turtles in Hawaii with implications for conservation. *Conserv. Genet.* **17**, 207–220. 10.1007/s10592-015-0772-7 (2015).

[22] Gong, S. P., Fritz, U., Vamberger, M., Gao, Y. C. & Farkas, B. Disentangling the *Pelodiscus axenaria* complex, with the description of a new Chinese species and neotype designation for *P. axenaria* (Zhou, Zhang & Fang, 1991). *Zootaxa* **5125**, 131–143. 10.11646/zootaxa.5125.2.2 (2022). 10.11646/zootaxa.5125.2.2 36101223

[23] Parham, J. F., Feldman, C. R. & Boore, J. L. The complete mitochondrial genome of the enigmatic bigheaded turtle (*Platysternon*): description of unusual genomic features and the reconciliation of phylogenetic hypotheses based on mitochondrial and nuclear DNA. *BMC Evol. Biol.* **6**, 11–11. 10.1186/1471-2148-6-11 (2006). 16464263 10.1186/1471-2148-6-11 PMC1403801

[24] Gong, S. P. et al. Genomic analyses reveal three phylogenetic species and their evolutionary histories in the big-headed turtle. *iScience* **26**, 107343. 10.1016/j.isci.2023.107343 (2023). 37539035 10.1016/j.isci.2023.107343 PMC10393795

[25] Duchene, S. et al. Marine turtle mitogenome phylogenetics and evolution. *Mol. Phylogenet. Evol.* **65**, 241–250. 10.1016/j.ympev.2012.06.010 (2012). 22750111 10.1016/j.ympev.2012.06.010

[26] Poulakakis, N. et al. Colonization history of Galapagos giant tortoises: insights from mitogenomes support the progression rule. *J. Zool. Syst. Evol. Res.* **58**, 1262–1275. 10.1111/jzs.12387 (2020).

[27] Montaña-Lozano, P., Balaguera-Reina, S. A. & Prada-Quiroga, C. F. Comparative analysis of codon usage of mitochondrial genomes provides evolutionary insights into reptiles. *Gene* **851**, 146999. 10.1016/j.gene.2022.146999 (2023). 36309241 10.1016/j.gene.2022.146999

[28] Montaña-Lozano, P. et al. Comparative genomic analysis of vertebrate mitochondrial reveals a differential of rearrangements rate between taxonomic class. *Sci. Rep.* **12**, 1–13. 10.1038/s41598-022-09512-2 (2022). 35361853 10.1038/s41598-022-09512-2 PMC8971445

[29] Duchêne, S., Archer, F. I., Vilstrup, J., Caballero, S. & Morin, P. A. Mitogenome phylogenetics: the impact of using single regions and partitioning schemes on topology, substitution rate and divergence time Estimation. *PLoS ONE* **6**, 138. 10.1371/journal.pone.0027138 (2011). 10.1371/journal.pone.0027138 PMC3206919 22073275

[30] Liedigk, R., Roos, C., Brameier, M. & Zinner, D. Mitogenomics of the old world monkey tribe Papionini. *BMC Evol. Biol.* **14**, 1–12. 10.1186/s12862-014-0176-1 (2014). 10.1186/s12862-014-0176-1 PMC4169223 25209564

[31] Huang, X. Q. & Madan, A. CAP3: A DNA sequence assembly program. *Genome Res.* **9**, 868–877. 10.1101/gr.9.9.868 (1999). 10508846 10.1101/gr.9.9.868 PMC310812

[32] Peterson, B. K., Weber, J. N., Kay, E. H., Fisher, H. S. & Hoekstra, H. E. Double digest RADseq: an inexpensive method for de Novo SNP discovery and genotyping in model and non-model species. *PLoS ONE* **7**, 135. 10.1371/journal.pone.0037135 (2012). 10.1371/journal.pone.0037135 PMC3365034 22675423

[33] Bankevich, A. et al. SPAdes: A new genome assembly algorithm and its applications to single-cell sequencing. *J. Comput. Biol.* **19**, 455–477. 10.1089/CMB.2012.0021 (2012). 22506599 10.1089/cmb.2012.0021 PMC3342519

[34] Lowe, T. M. & Eddy, S. R. tRNAscan-SE: a program for improved detection of transfer RNA genes in genomic sequence. *Nucleic Acids Res.* **25**, 955–964. 10.1093/nar/25.5.955 (1997). 9023104 10.1093/nar/25.5.955 PMC146525

[35] Greiner, S., Lehwark, P. & Bock, R. OrganellarGenomeDRAW (OGDRAW) version 1.3.1: expanded toolkit for the graphical visualization of organellar genomes. *Nucleic Acids Res.* **47**, W59–W64. 10.1093/nar/gkz238 (2019). 30949694 10.1093/nar/gkz238 PMC6602502

[36] Kumar, S., Stecher, G., Li, M., Knyaz, C. & Tamura, K. MEGA X: molecular evolutionary genetics analysis across computing platforms. *Mol. Biol. Evol.* **35**, 1547–1549. 10.1093/MOLBEV/MSY096 (2018). 29722887 10.1093/molbev/msy096 PMC5967553

[37] Perna, N. T. & Kocher, T. D. Patterns of nucleotide composition at fourfold degenerate sites of animal mitochondrial genomes. *J. Mol. Evol.* **41**, 353–358. 10.1007/BF00186547 (1995). 7563121 10.1007/BF00186547

[38] Benson, G. Tandem repeats finder: a program to analyze DNA sequences. *Nucleic Acids Res.* **27**, 573–580. 10.1093/nar/27.2.573 (1999). 9862982 10.1093/nar/27.2.573 PMC148217

[39] Walberg, M. W. & Clayton, D. A. Sequence and properties of the human KB cell and mouse L cell D-loop regions of mitochondrial DNA. *Nucleic Acids Res.* **9**, 5411–5421. 10.1093/nar/9.20.5411 (1981). 7301592 10.1093/nar/9.20.5411 PMC327529

[40] Sbisa, E., Tanzariello, F., Reyes, A., Pesole, G. & Saccone, C. Mammalian mitochondrial D-loop region structural analysis: identification of new conserved sequences and their functional and evolutionary implications. *Gene* **205**, 125–140. 10.1016/s0378-1119(97)00404-6 (1997). 9461386 10.1016/s0378-1119(97)00404-6

[41] Dauzery, E. & Randi, E. The mitochondrial control region of cervidae: evolutionary patterns and phylogenetic content. *Mol. Biol. Evol.* **14**, 1154–1166. 10.1093/oxfordjournals.molbev.a025725 (1997). 9364773 10.1093/oxfordjournals.molbev.a025725

[42] Bailey, T. L. & Gribskov, M. Combining evidence using p-values: application to sequence homology searches. *Bioinformatics* **14**, 48–54. 10.1093/bioinformatics/14.1.48 (1998). 9520501 10.1093/bioinformatics/14.1.48

[43] Zhang, Z. KaKs_Calculator 3.0: calculating selective pressure on coding and non-coding sequences. *Genom. Proteom. Bioinf.* **20**, 536–540. 10.1016/j.gpb.2021.12.002 (2022). 10.1016/j.gpb.2021.12.002 PMC9801026 34990803

[44] Yang, Z. PAML 4: phylogenetic analysis by maximum likelihood. *Mol. Biol. Evol.* **24**, 1586–1591. 10.1093/molbev/msm088 (2007). 17483113 10.1093/molbev/msm088

[45] Álvarez-Carretero, S., Kapli, P. & Yang, Z. Beginner’s guide on the use of PAML to detect positive selection. *Mol. Biol. Evol.* **40**, 1–18. 10.1093/molbev/msad041 (2023). 10.1093/molbev/msad041 PMC10127084 37096789

[46] Yang, Z., Wong, W. S. W. & Nielsen, R. Bayes empirical Bayes inference of amino acid sites under positive selection. *Mol. Biol. Evol.* **22**, 1107–1118. 10.1093/molbev/msi097 (2005). 15689528 10.1093/molbev/msi097

[47] Zhang, D. et al. PhyloSuite: an integrated and scalable desktop platform for streamlined molecular sequence data management and evolutionary phylogenetics studies. *Mol. Ecol. Resour.* **20**, 348–355. 10.1111/1755-0998.13096 (2020). 31599058 10.1111/1755-0998.13096

[48] Castresana, J. Selection of conserved blocks from multiple alignments for their use in phylogenetic analysis. *Mol. Biol. Evol.* **17**, 540–552. 10.1093/oxfordjournals.molbev.a026334 (2000). 10742046 10.1093/oxfordjournals.molbev.a026334

[49] Hall, A. T. BIOEDIT: A user-friendly biological sequence alignment editor and analysis program for windows 95/98/NT. *Nucleic Acids Symp. Ser.* **41**, 95–98. 10.14601/PHYTOPATHOL_MEDITERR-14998U1.29 (1999).

[50] Ronquist, F. et al. MrBayes 3.2: efficient bayesian phylogenetic inference and model choice across a large model space. *Syst. Biol.* **61**, 539–542. 10.1093/sysbio/sys029 (2012). 22357727 10.1093/sysbio/sys029 PMC3329765

[51] Lanfear, R., Frandsen, P. B., Wright, A. M., Senfeld, T. & Calcott, B. Partitionfinder 2: new methods for selecting partitioned models of evolution for molecular and morphological phylogenetic analyses. *Mol. Biol. Evol.* **34**, 772–773. 10.1093/molbev/msw260 (2017). 28013191 10.1093/molbev/msw260

[52] Nguyen, L. T., Schmidt, H. A., Von Haeseler, A. & Minh, B. Q. IQ-TREE: A fast and effective stochastic algorithm for estimating maximum-likelihood phylogenies. *Mol. Biol. Evol.* **32**, 268–274. 10.1093/molbev/msu300 (2015). 25371430 10.1093/molbev/msu300 PMC4271533

[53] Stamatakis, A. RAxML version 8: A tool for phylogenetic analysis and post-analysis of large phylogenies. *Bioinformatics* **30**, 1312–1313. 10.1093/bioinformatics/btu033 (2014). 24451623 10.1093/bioinformatics/btu033 PMC3998144

[54] Shimodaira, H. An approximately unbiased test of phylogenetic tree selection. *Syst. Biol.* **51**, 492–508. 10.1080/10635150290069913 (2002). 12079646 10.1080/10635150290069913

[55] Bouckaert, R. et al. BEAST 2: A software platform for bayesian evolutionary analysis. *PLoS Comput. Biol.* **10**, 1–6. 10.1371/journal.pcbi.1003537 (2014). 10.1371/journal.pcbi.1003537 PMC3985171 24722319

[56] Bouckaert, R. R., Drummond, A. J. & bModelTest Bayesian phylogenetic site model averaging and model comparison. *BMC Evol. Biol.* **17**, 1–11. 10.1186/S12862-017-0890-6 (2017). 28166715 10.1186/s12862-017-0890-6 PMC5294809

[57] Joyce, W. G., Parham, J. F., Lyson, T. R., Warnock, R. C. M. & Donoghue, P. C. J. A divergence dating analysis of turtles using fossil calibrations: an example of best practices. *J. Paleontol.* **87**, 612–634. 10.1666/12-149 (2013).

[58] Massonne, T., Augustin, F. J., Matzke, A. T. & Böhme, M. A new Cryptodire from the eocene of the Na Duong basin (northern Vietnam) sheds new light on *Pan-Trionychidae* from Southeast Asia. *J. Syst. Paleontol.* **21**, 25. 10.1080/14772019.2023.2217505 (2023).

[59] Jühling, F. et al. Improved systematic tRNA gene annotation allows new insights into the evolution of mitochondrial tRNA structures and into the mechanisms of mitochondrial genome rearrangements. *Nucleic Acids Res.* **40**, 2833–2845. 10.1093/nar/gkr1131 (2012). 22139921 10.1093/nar/gkr1131 PMC3326299

[60] Le, M. et al. A phylogeny of softshell turtles (Testudines: Trionychidae) with reference to the taxonomic status of the critically endangered, giant softshell turtle, *Rafetus swinhoei*. *Org. Divers. Evol.* **14**, 279–293. 10.1007/s13127-014-0169-3 (2014).

[61] Min, X. J. & Hickey, D. A. DNA asymmetric strand bias affects the amino acid composition of mitochondrial proteins. *DNA Res.* **14**, 201–206. 10.1093/dnares/dsm019 (2007). 17974594 10.1093/dnares/dsm019 PMC2779903

[62] Saccone, C., De Giorgi, C., Gissi, C., Pesole, G. & Reyes, A. Evolutionary genomics in metazoa: the mitochondrial DNA as a model system. *Gene* **238**, 195–209. 10.1016/S0378-1119(99)00270-X (1999). 10570997 10.1016/s0378-1119(99)00270-x

[63] Reyes, A., Gissi, C., Pesole, G. & Saccone, C. Asymmetrical directional mutation pressure in the mitochondrial genome of mammals. *Mol. Biol. Evol.* **15**, 957–966. 10.1093/oxfordjournals.molbev.a026011 (1998). 9718723 10.1093/oxfordjournals.molbev.a026011

[64] Xia, X. H. Mutation and selection on the anticodon of tRNA genes in vertebrate mitochondrial genomes. *Gene* **345**, 13–20. 10.1016/j.gene.2004.11.019 (2005). 15716092 10.1016/j.gene.2004.11.019

[65] Saccone, C., Pesole, G. & Sbisá, E. The main regulatory region of mammalian mitochondrial DNA: Structure-function model and evolutionary pattern. *J. Mol. Evol.* **33**, 83–91. 10.1007/BF02100199 (1991). 1909377 10.1007/BF02100199

[66] Tikochinski, Y. et al. Mitochondrial DNA short tandem repeats unveil hidden population structuring and migration routes of an endangered marine turtle. *Aquat. Conserv. Mar. Freshw. Ecosyst.* **28**, 788–797. 10.1002/aqc.2908 (2018).

[67] Taanman, J. W. The mitochondrial genome: structure, transcription, translation and replication. *Biochim. Biophys. Acta Bioenerg.* **1410**, 103–123. 10.1016/S0005-2728(98)00161-3 (1999). 10.1016/s0005-2728(98)00161-3 10076021

[68] Randi, E. & Lucchini, V. Organization and evolution of the mitochondrial DNA control region in the avian genus *Alectoris*. *J. Mol. Evol.* **47**, 449–462. 10.1007/PL00006402 (1998). 9767690 10.1007/pl00006402

[69] Liu, H. Z., Tzeng, C. S. & Teng, H. Y. Sequence variations in the mitochondrial DNA control region and their implications for the phylogeny of the Cypriniformes. *Can. J. Zool.* **80**, 569–581. 10.1139/z02-035 (2002).

[70] Jiang, L. C. et al. Complete mitochondrial genome sequence of the Himalayan Griffon, *Gyps himalayensis* (Accipitriformes: Accipitridae): Sequence, structure, and phylogenetic analyses. *Ecol. Evol.* **9**, 8813–8828. 10.1002/ece3.5433 (2019). 31410282 10.1002/ece3.5433 PMC6686361

[71] Song, X. H. et al. The complete mitochondrial genome of *Accipiter virgatus* and evolutionary history of the pseudo-control regions in Falconiformes. *Biochem. Syst. Ecol.* **58**, 75–84. 10.1016/j.bse.2014.10.013 (2015).

[72] Horner, D. S. et al. Phylogenetic analyses of complete mitochondrial genome sequences suggest a basal divergence of the enigmatic rodent *Anomalurus*. *BMC Evol. Biol.* **7**, 1–12. 10.1186/1471-2148-7-16 (2007). 17288612 10.1186/1471-2148-7-16 PMC1802082

[73] Wolstenholme, D. R. Animal mitochondrial DNA: structure and evolution. *Int. Rev. Cytol.* **141**, 173–216. 10.1016/S0074-7696(08)62066-5 (1992). 1452431 10.1016/s0074-7696(08)62066-5

[74] Larizza, A., Pesole, G., Reyes, A., Sbisà, E. & Saccone, C. Lineage specificity of the evolutionary dynamics of the MtDNA D-loop region in rodents. *J. Mol. Evol.* **54**, 145–155. 10.1007/s00239-001-0063-4 (2002). 11821908 10.1007/s00239-001-0063-4

[75] Dillon, M. C. & Wright, J. M. Nucleotide sequence of the D-loop region of the sperm Whale (*Physeter macrocephalus*) mitochondrial genome. *Mol. Biol. Evol.* **10**, 296–305. 10.1093/oxfordjournals.molbev.a040005 (1993). 8487632 10.1093/oxfordjournals.molbev.a040005

[76] Southern, Š. O., Southern, P. J. & Dizon, A. E. Molecular characterization of a cloned Dolphin mitochondrial genome. *J. Mol. Evol.* **28**, 32–42. 10.1007/BF02143495 (1988). 3148740 10.1007/BF02143495

[77] Wood, N. J. & Phua, S. H. Variation in the control region sequence of the sheep mitochondrial genome. *Anim. Genet.* **27**, 25–33. 10.1111/j.1365-2052.1996.tb01173.x (1996). 8624033 10.1111/j.1365-2052.1996.tb01173.x

[78] Shen, Y. Y., Shi, P., Sun, Y. B. & Zhang, Y. P. Relaxation of selective constraints on avian mitochondrial DNA following the degeneration of flight ability. *Genome Res.* **19**, 1760–1765. 10.1101/gr.093138.109 (2009). 19617397 10.1101/gr.093138.109 PMC2765268

[79] Sun, Y. B., Shen, Y. Y., Irwin, D. M. & Zhang, Y. P. Evaluating the roles of energetic functional constraints on teleost mitochondrial-encoded protein evolution. *Mol. Biol. Evol.* **28**, 39–44. 10.1093/molbev/msq256 (2011). 20924083 10.1093/molbev/msq256

[80] Chong, R. A. & Mueller, R. Low metabolic rates in salamanders are correlated with weak selective constraints on mitochondrial genes. *Evolution* **67**, 894–899. 10.1111/j.1558-5646.2012.01830.x (2013). 23461338 10.1111/j.1558-5646.2012.01830.x

[81] Castellana, S., Vicario, S. & Saccone, C. Evolutionary patterns of the mitochondrial genome in metazoa: exploring the role of mutation and selection in mitochondrial protein-coding genes. *Genome Biol. Evol.* **3**, 1067–1079. 10.1093/gbe/evr040 (2011). 21551352 10.1093/gbe/evr040 PMC3229188

[82] Fonseca, M. M. & Harris, D. J. Relationship between mitochondrial gene rearrangements and stability of the origin of light strand replication. *Genet. Mol. Biol.* **31**, 566–574. 10.1590/S1415-47572008000300027 (2008).

[83] Sun, S. E., Li, Q., Kong, L. F. & Yu, H. Limited locomotive ability relaxed selective constraints on molluscs mitochondrial genomes. *Sci. Rep.* **7**, 1. 10.1038/s41598-017-11117-z (2017). 28878314 10.1038/s41598-017-11117-z PMC5587578

[84] Stuckas, H. & Fritz, U. Identity of *Pelodiscus sinensis* revealed by DNA sequences of an approximately 180-year-old type specimen and a taxonomic reappraisal of *Pelodiscus* species (Testudines: Trionychidae). *J. Zool. Syst. Evol. Res.* **49**, 335–339. 10.1111/j.1439-0469.2011.00632.x (2011).

[85] Fritz, U. et al. The world’s economically most important Chelonians represent a diverse species complex (Testudines: Trionychidae: *Pelodiscus*). *Org. Divers. Evol.* **10**, 227–242. 10.1007/s13127-010-0007-1 (2010).

[86] Praschag, P. et al. Geoemyda silvatica, an enigmatic turtle of the Geoemydidae (Reptilia: Testudines), represents a distinct genus. *Org. Divers. Evol.* **6**, 151–162. 10.1016/j.ode.2005.10.001 (2006).

[87] Vargas-Ramirez, M. et al. Deep genealogical lineages in the widely distributed African helmeted terrapin: evidence from mitochondrial and nuclear DNA (Testudines: Pelomedusidae: *Pelomedusa subrufa*). *Mol. Phylogenet. Evol.* **56**, 428–440. 10.1016/j.ympev.2010.03.019 (2010). 20332032 10.1016/j.ympev.2010.03.019

[88] Iverson, J. B., Le, M. & Ingram, C. Molecular phylogenetics of the mud and musk turtle family Kinosternidae. *Mol. Phylogenet. Evol.* **69**, 929–939. 10.1016/j.ympev.2013.06.011 (2013). 23823161 10.1016/j.ympev.2013.06.011

[89] Gong, Y. A. et al. A new species of the genus Pelodiscus Fitzinger, 1835 (Testudines: Trionychidae) from Huangshan, Anhui, China. *Zootaxa* **5060**, 137–145. 10.11646/zootaxa.5060.1.7 (2021). 34811179 10.11646/zootaxa.5060.1.7

[90] Nabhan, A. R. & Sarkar, I. N. The impact of taxon sampling on phylogenetic inference: A review of two decades of controversy. *Brief. Bioinform.* **13**, 122–134. 10.1093/bib/bbr014 (2012). 21436145 10.1093/bib/bbr014 PMC3251835

[91] Mayden, R. L. et al. Reconstructing the phylogenetic relationships of the Earth’s most diverse clade of freshwater fishes-order Cypriniformes (Actinopterygii: Ostariophysi): A case study using multiple nuclear loci and the mitochondrial genome. *Mol. Phylogenet. Evol.* **51**, 500–514. 10.1016/j.ympev.2008.12.015 (2009). 19141325 10.1016/j.ympev.2008.12.015

[92] Prevosti, F. J. & Chemisquy, M. A. The impact of missing data on real morphological phylogenies: influence of the number and distribution of missing entries. *Cladistics* **26**, 326–339. 10.1111/j.1096-0031.2009.00289.x (2010). 34875786 10.1111/j.1096-0031.2009.00289.x

[93] Wolsan, M. & Sato, J. J. Effects of data incompleteness on the relative performance of parsimony and bayesian approaches in a supermatrix phylogenetic reconstruction of Mustelidae and Procyonidae (Carnivora). *Cladistics* **26**, 168–194. 10.1111/j.1096-0031.2009.00281.x (2010). 34875759 10.1111/j.1096-0031.2009.00281.x

[94] Wiens, J. J. & Morrill, M. C. Missing data in phylogenetic analysis: reconciling results from simulations and empirical data. *Syst. Biol.* **60**, 719–731. 10.1093/sysbio/syr025 (2011). 21447483 10.1093/sysbio/syr025

[95] Chojnowski, J. L., Kimball, R. T. & Braun, E. L. Introns outperform exons in analyses of basal avian phylogeny using clathrin heavy chain genes. *Gene* **410**, 89–96. 10.1016/j.gene.2007.11.016 (2008). 18191344 10.1016/j.gene.2007.11.016

[96] Kimball, R. T. & Braun, E. L. Does more sequence data improve estimates of Galliform phylogeny? Analyses of a rapid radiation using a complete data matrix. *PeerJ* **2014**, 361. 10.7717/peerj.361 (2014). 10.7717/peerj.361 PMC4006227 24795852

[97] Brown, J. M. & Lemmon, A. R. The importance of data partitioning and the utility of Bayes factors in bayesian phylogenetics. *Syst. Biol.* **56**, 643–655. 10.1080/10635150701546249 (2007). 17661232 10.1080/10635150701546249

[98] Castoe, T. A., Doan, T. M. & Parkinson, C. L. Data partitions and complex models in bayesian analysis: the phylogeny of gymnophthalmid lizards. *Syst. Biol.* **53**, 448–469. 10.1080/10635150490445797 (2004). 15503673 10.1080/10635150490445797

[99] Hren, M. T. et al. Terrestrial cooling in Northern Europe during the Eocene–Oligocene transition. *Proc. Natl. Acad. Sci. U.S.A.* **110**, 7562–7567. 10.1073/pnas.1210930110 (2013). 23610424 10.1073/pnas.1210930110 PMC3651463

[100] Thomson, R. C., Spinks, P. Q. & Bradley Shaffer, H. A global phylogeny of turtles reveals a burst of climate-associated diversification on continental margins. *Proc. Natl. Acad. Sci. U.S.A.* **118**, 1–10. 10.1073/pnas.2012215118 (2021). 10.1073/pnas.2012215118 PMC7896334 33558231

[101] Barley, A. J., Spinks, P. Q., Thomson, R. C. & Shaffer, H. B. Fourteen nuclear genes provide phylogenetic resolution for difficult nodes in the turtle tree of life. *Mol. Phylogenet. Evol.* **55**, 1189–1194. 10.1016/j.ympev.2009.11.005 (2010). 19913628 10.1016/j.ympev.2009.11.005

[102] Wiens, J. J., Kuczynski, C. A. & Stephens, P. R. Discordant mitochondrial and nuclear gene phylogenies in emydid turtles: implications for speciation and conservation. *Biol. J. Linn. Soc.* **99**, 445–461. 10.1111/j.1095-8312.2009.01342.x (2010).

[103] Spinks, P. Q. et al. Species boundaries and phylogenetic relationships in the critically endangered Asian box turtle genus *Cuora*. *Mol. Phylogenet. Evol.* **63**, 656–667. 10.1016/j.ympev.2012.02.014 (2012). 22649793 10.1016/j.ympev.2012.02.014

[104] Blair, C. Organellar DNA continues to provide a rich source of information in the genomics era. *Mol. Ecol.* **32**, 2144–2150. 10.1111/mec.16872 (2023). 36727263 10.1111/mec.16872

[105] Zhang, L. et al. Complete mitochondrial genomes reveal robust phylogenetic signals and evidence of positive selection in horseshoe bats. *BMC Ecol. Evol.* **21**, 1–15. 10.1186/s12862-021-01926-2 (2021). 34732135 10.1186/s12862-021-01926-2 PMC8565063

[106] Morgan, B. et al. Long-read sequencing data reveals dynamic evolution of mitochondrial genome size and the phylogenetic utility of mitochondrial DNA in Hercules beetles (Dynastes; Scarabaeidae). *Genome Biol. Evol.* **14**, 1–14. 10.1093/gbe/evac147 (2022). 10.1093/gbe/evac147 PMC9576211 36173740

[107] Havird, J. C. & Santos, S. R. Performance of single and concatenated sets of mitochondrial genes at inferring metazoan relationships relative to full mitogenome data. *PLoS ONE* **9**, 1–10. 10.1371/journal.pone.0084080 (2014). 10.1371/journal.pone.0084080 PMC3891902 24454717

[108] Wiens, J. J., Fetzner, J. W., Parkinson, C. L. & Reeder, T. W. Hylid frog phylogeny and sampling strategies for speciose clades. *Syst. Biol.* **54**, 719–748. 10.1080/10635150500234625 (2005). 16243760 10.1080/10635150500234625

