# Supplementary material for: Comparative analysis of the mitochondrial genomes of the soft-shelled turtles Palea steindachneri and Pelodiscus axenaria and phylogenetic implications for Trionychia
Source: Sci Rep. 2025 Feb 28;15:7138. doi: 10.1038/s41598-025-90985-2 (PMC11871352; doi:10.1038/s41598-025-90985-2)
Supplement: Supplementary file 1 — Supplementary Material 1 [file 41598_2025_90985_MOESM1_ESM.docx]

**Supplementary Materials**

# Comparative analysis of the mitochondrial genomes of the soft-shelled turtles *Palea steindachneri* and *Pelodiscus axenaria*, and phylogenetic implications for Trionychia

# Chen Chen^a^ · Liqin Ji^a^ · Guiyun Huang^b^ · Xiaoli Liu^a^ · Haigang Chen^a^ · Yakun Wang^a^ · Lingyun Yu^a^ · Yihui Liu^a^ · Xiaoyou Hong^a^ · Chengqing Wei^a^ · Congcong Wu^a^ · Laifu Luo^a^ · Xinping Zhu^a*^ · Wei Li^a*^

# C. Chen^a^ · L.Q. Ji^a^ · G.Y. Huang^b^ · X.L. Liu^a^ · H.G. Chen^a^ · Y.K. Wang^a^ · L.Y. Yu^a^ · Y.H. Liu^a^ · X.Y. Hong^a^ · C.Q. Wei^a^ · C.C. Wu^a^ · L.F. Luo^a^ · X.P. Zhu^a^ (✉) · W. Li^a^ (✉)

a. Key Laboratory of Tropical and Subtropical Fishery Resources Application and Cultivation, Ministry of Agriculture and Rural Affairs, Pearl River Fisheries Research Institute, Chinese Academy of Fishery Sciences, Guangzhou 510380, China

b. Agro-Tech Extension Center of Guangdong Province，Department of Agriculture of Guangdong Province, Guangzhou, China

Email: [zhuxinping_1964@163.com](mailto:zhuxinping_1964@163.com); [664061430@qq.com](mailto:664061430@qq.com)

Table S1. PCR primers and reaction conditions for the Wattle-necked soft-shelled turtle, *Palea steindachneri,­­* mitochondrial genome.

| Primers | Primer Sequence | Size (bp) | *Ta* (℃) | Product’s Target Regions |
| --- | --- | --- | --- | --- |
| 12s(e)-F | AGTGAAAATGCCCTAAAAGTCACATC | 1869 | 54 | 12S, trnV, partial 16S |
| 12s(e)-R | ATACTTATTGTTGCTAGGGGCTATGT |  |  |  |
| 16s(g)-F | AATAACAGATGGGGTAAGTCGTAACA | 2018 | 56 | partial 12S, trnV, 16S, trnL2, partial *nad*1 |
| 16s(g)-R | GTGAAGAAGGCTACAGCAATTAAGAT |  |  |  |
| 16Co(g)-F | GAGTTCAGACCGGAGCAATCCA | 3136 | 54 | partial 16S, trnL2, *nad*1, trnI, trnQ, trnM, *nad*2, trnW, trnA, trnN, trnC, trnY, partial *cox*1 |
| 16Co(g)-R | CAGTTCCTGCGCCTGTTTCAAT |  |  |  |
| COI(d)-F | CTACATGGTTTGATAAGAAGGGGAGT | 1718 | 56 | *cox*1, trnS2, trnD |
| COI(d)-R | ATTGGTGATATTGCGTCTTGAAATCC |  |  |  |
| COI-COIII-F | CACTACACCAAACCTGAACCAAAGTA | 2551 | 62 | partial *cox*1, trnS2, trnD, *cox*2, trnK, *atp*8, *atp*6, partial *cox*3 |
| COI-COIII-R | GATTGTGAATGGTGCTTCGTAGTATTC |  |  |  |
| COIII(f)-F | CACACAACTATCAATGAACATAGCAC | 1152 | 56 | partial *atp*6, *cox*3, partial trnG |
| COIII(f)-R | TGAACTGAAATTGAATGATTGGAAGT |  |  |  |
| COND-F | CTACAAGCCATAGAATACTACGAAGCAC | 2785 | 66 | partial *cox*3, trnG, *nad*3, trnR, *nad*4L, *nad*4, trnH, trnS1, trnL1, partial *nad*5 |
| COND-R | TTCAAGATCAGTTGGTGATGATTGATTC |  |  |  |
| ND5(m)-F | AATCTCCTTATAAACCGAGAAGGT | 1898 | 54 | parital trnS1, trnL1, *nad*5, partial *nad*6 |
| ND5(m)-R | AGATTTAGTTCGTGGTTTGGCT |  |  |  |
| NdCyt-F | ATCATTGCAGGACTACTAATCTCATCA | 1082 | 52 | partial *nad*5, *nad*6, trnE, partial *cytb* |
| NdCyt-R | ATTTCATCAGATGGAGATGTTAGATGGA |  |  |  |
| Cytb(g)-F | GTCAACGCCACAGAATAAGC | 1491 | 54 | partial *nad*6, trnE, *cytb*, trnT |
| Cytb(g)-R | ATTCCGGTTTTGGGGATCGG |  |  |  |
| Cyt12(f)-F | GCCAACCTTGCAGTACTAACATGAAT | 2138 | 54 | partical *cytb*, trnT, trnP, Control Region, trnF, partial 12S |
| Cyt12(f)-R | TCTAATCCCAGTTTGTGTCTTAGCGA |  |  |  |

Table S2. Trionychia and outgroup species used in phylogenetic analysis and substitution rates of protein-coding genes in this study, with their accession numbers.

| Species | Accession No. | Length | References |
| --- | --- | --- | --- |
| *Amyda cartilaginea* | NC_054232.1/MT039230.1 | 16763 | Cui, Rao, and Zhang, 2020 |
| *Apalone ferox* | KX882744.1 | 16817 | Yu et al., 2019 |
| *Apalone ferox* | NC_014054.1/FJ890514.1 | 16866 | Li et al., 2017 |
| *Apalone spinifera* | KX882745.1 | 16756 | Yu et al., 2019 |
| *Apalone spinifera* | NC_021371.1/JF966197.1 | 16749 | Li et al., 2017 |
| *Carettochelys insculpta* | NC_014048.1/FJ862792.1 | 16439 | Li et al., 2010 |
| *Chitra indica* | NC_026028.1/JQ406951.1 | 16726 | Li et al., 2017 |
| *Chitra vandijki* | NC_053617.1 | 16614 | Chen et al., 2021 |
| *Dogania subplana* | NC_002780.1/AF366350.1 | 17289 | Unpublished |
| *Lissemys punctata* | NC_012414.1/EF050073.1 | 16490 | Unpublished |
| *Lissemys scutata* | NC_025494.1/JQ361816.1 | 16512 | Li et al., 2017 |
| *Mauremys mutica* | NC_009330.1/DQ453753.1 | 16609 | Unpublished |
| *Mauremys reevesii* | FJ469674.1 | 16783 | Unpublished |
| *Nilssonia nigricans* | NC_039559.1/MG383833.1 | 16796 | Kundu et al., 2018 |
| *Palea steindachneri* | KX882746.1 | 16875 | Unpublished |
| *Palea steindachneri* | NC_013841.1/FJ541030.1 | 17243 | Li et al., 2017 |
| *Palea steindachneri* | OL405264.1 | 16811 | This study |
| *Pelochelys cantorii* | JN016747.1 | 17612 | Chen et al., 2013 |
| *Pelochelys cantorii* | KT962834.1 | 17424 | Zhang et al., 2018 |
| *Pelochelys cantorii* | NC_015825.1/JN016746.1 | 17499 | Chen et al., 2013 |
| *Pelodiscus axenaria* | OR805132.1 | 17143 | This study |
| *Pelodiscus maackii* | OK377340.1 | 16258 | Baek et al., 2022 |
| *Pelodiscus sinensis* | AY687385.1 | 17364 | Unpublished |
| *Pelodiscus maackii* | AY962573.1^*^ | 17042 | Jung et al., 2006 |
| *Pelodiscus sinensis* | MG431983.1 | 17145 | Zhang et al., 2019 |
| *Rafetus swinhoei* | KU997642.1 | 16778 | Unpublished |
| *Rafetus swinhoei* | MT039229.1 | 16812 | Unpublished |
| *Rafetus swinhoei* | NC_017901.1/HQ709384.1 | 16990 | Li et al., 2017 |
| *Rafetus swinhoei* | OK323959.1 | 16622 | Unpublished |
| *Trionyx triunguis* | NC_012833.1/AB477345.1 | 16590 | Amer and Kumazawa et al., 2009 |

^*^ Species name of AY962573.1 was revised by Fritz et al. (2010).

Table S3. Codon usage of PCGs in the mitochondrial genomes of *Palea steindachneri* (OL405264) and *Pelodiscus axenaria* (OR805132).

| AA | Codon | *Palea steindachneri* | *Pelodiscus axenaria* |
| --- | --- | --- | --- |
| Ala | GCU | 58 | 49 |
| Ala | GCC | 92 | 95 |
| Ala | GCA | 82 | 87 |
| Ala | GCG | 2 | 3 |
| Arg | CGU | 7 | 7 |
| Arg | CGC | 11 | 11 |
| Arg | CGA | 46 | 50 |
| Arg | CGG | 4 | 1 |
| Asn | AAU | 47 | 62 |
| Asn | AAC | 117 | 107 |
| Asp | GAU | 22 | 25 |
| Asp | GAC | 42 | 36 |
| Cys | UGU | 9 | 14 |
| Cys | UGC | 17 | 14 |
| Gln | CAA | 96 | 98 |
| Gln | CAG | 7 | 5 |
| Glu | GAA | 78 | 77 |
| Glu | GAG | 5 | 8 |
| Gly | GGU | 29 | 48 |
| Gly | GGC | 59 | 45 |
| Gly | GGA | 78 | 79 |
| Gly | GGG | 36 | 31 |
| His | CAU | 38 | 41 |
| His | CAC | 66 | 60 |
| Ile | AUU | 147 | 177 |
| Ile | AUC | 163 | 153 |
| Leu | UUA | 165 | 227 |
| Leu | UUG | 14 | 20 |
| Leu | CUU | 56 | 64 |
| Leu | CUC | 60 | 58 |
| Leu | CUA | 277 | 213 |
| Leu | CUG | 25 | 17 |
| Lys | AAA | 91 | 86 |
| Lys | AAG | 0 | 3 |
| Met | AUA | 209 | 214 |
| Met | AUG | 30 | 33 |
| Phe | UUU | 88 | 104 |
| Phe | UUC | 126 | 110 |
| Pro | CCU | 30 | 38 |
| Pro | CCC | 44 | 33 |
| Pro | CCA | 133 | 132 |
| Pro | CCG | 3 | 3 |
| Ser | UCU | 37 | 29 |
| Ser | UCC | 47 | 52 |
| Ser | UCA | 142 | 137 |
| Ser | UCG | 2 | 5 |
| Ser | AGU | 14 | 15 |
| Ser | AGC | 33 | 36 |
| Thr | ACU | 56 | 55 |
| Thr | ACC | 151 | 128 |
| Thr | ACA | 184 | 186 |
| Thr | ACG | 4 | 5 |
| Trp | UGA | 100 | 96 |
| Trp | UGG | 9 | 13 |
| Tyr | UAU | 50 | 55 |
| Tyr | UAC | 73 | 69 |
| Val | GUU | 34 | 40 |
| Val | GUC | 26 | 20 |
| Val | GUA | 85 | 76 |
| Val | GUG | 16 | 17 |

Table S4. Likelihood ratio test (LRT) for positive selection under site models of the 13 mitochondrial PCGs genes of 17 Trionychia species performed on PAML.

| PCGs | Model comparison | *ℓ*^a^ | Free parameters^b^ | df | 2Δ*ℓ*^c^ | Positively selected codons^d^ | posterior mean ± SE for ω | PP (ω > 1) |
| --- | --- | --- | --- | --- | --- | --- | --- | --- |
| *atp*6 | M0 vs. M1a (one-ratio vs. nearly neutral) | *ℓ*_0_ = -4656.81 | 37 vs. 38 | 1 | **100.13** | —— | —— | —— |
|  |  | *ℓ*_1_ = -4606.74 |  |  |  |  |  |  |
|  | M1a vs. M2a (nearly neutral vs. positive selection) | *ℓ*_0_ = -4606.74 | 38 vs. 40 | 2 | 0.00 |  |  |  |
|  |  | *ℓ*_1_ = -4606.74 |  |  |  |  |  |  |
|  | M7 vs. M8 (beta vs. beta&ω) | *ℓ*_0_ = -4528.25 | 38 vs. 40 | 2 | 0.00 |  |  |  |
|  |  | *ℓ*_1_ = -4528.26 |  |  |  |  |  |  |
| *atp*8 | M0 vs. M1a (one-ratio vs. nearly neutral) | *ℓ*_0_ = -1267.03 | 37 vs. 38 | 1 | **69.11** | —— | —— | —— |
|  |  | *ℓ*_1_ = -1232.47 |  |  |  |  |  |  |
|  | M1a vs. M2a (nearly neutral vs. positive selection) | *ℓ*_0_ = -1232.47 | 38 vs. 40 | 2 | 0.00 |  |  |  |
|  |  | *ℓ*_1_ = -1232.47 |  |  |  |  |  |  |
|  | M7 vs. M8 (beta vs. beta&ω) | *ℓ*_0_ = -1230.32 | 38 vs. 40 | 2 | 2.89 |  |  |  |
|  |  | *ℓ*_1_ = -1228.88 |  |  |  |  |  |  |
| *cox*1 | M0 vs. M1a (one-ratio vs. nearly neutral) | *ℓ*_0_ = -8277.44 | 37 vs. 38 | 1 | **32.99** | —— | —— | —— |
|  |  | *ℓ*_1_ = -8260.94 |  |  |  |  |  |  |
|  | M1a vs. M2a (nearly neutral vs. positive selection) | *ℓ*_0_ = -8260.94 | 38 vs. 40 | 2 | 0.00 |  |  |  |
|  |  | *ℓ*_1_ = -8260.94 |  |  |  |  |  |  |
|  | M7 vs. M8 (beta vs. beta&ω) | *ℓ*_0_ = -8203.45 | 38 vs. 40 | 2 | 5.39 |  |  |  |
|  |  | *ℓ*_1_ = -8200.76 |  |  |  |  |  |  |
| *cox*2 | M0 vs. M1a (one-ratio vs. nearly neutral) | *ℓ*_0_ = -3533.50 | 37 vs. 38 | 1 | **48.57** | —— | —— | —— |
|  |  | *ℓ*_1_ = -3509.22 |  |  |  |  |  |  |
|  | M1a vs. M2a (nearly neutral vs. positive selection) | *ℓ*_0_ = -3509.22 | 38 vs. 40 | 2 | 0.00 |  |  |  |
|  |  | *ℓ*_1_ = -3509.22 |  |  |  |  |  |  |
|  | M7 vs. M8 (beta vs. beta&ω) | *ℓ*_0_ = -3490.45 | 38 vs. 40 | 2 | **7.80** |  |  |  |
|  |  | *ℓ*_1_ = -3486.55 |  |  |  |  |  |  |
| *cox*3 | M0 vs. M1a (one-ratio vs. nearly neutral) | *ℓ*_0_ = -4235.41 | 37 vs. 38 | 1 | **124.08** | 216 M | 1.496 *±* 0.990 | 0.647 |
|  |  | *ℓ*_1_ = -4173.37 |  |  |  |  |  |  |
|  | M1a vs. M2a (nearly neutral vs. positive selection) | *ℓ*_0_ = -4173.37 | 38 vs. 40 | 2 | 0.00 |  |  |  |
|  |  | *ℓ*_1_ = -4173.37 |  |  |  |  |  |  |
|  | M7 vs. M8 (beta vs. beta&ω) | *ℓ*_0_ = -4129.13 | 38 vs. 40 | 2 | **18.86** |  |  |  |
|  |  | *ℓ*_1_ = -4119.70 |  |  |  |  |  |  |
| *cytb* | M0 vs. M1a (one-ratio vs. nearly neutral) | *ℓ*_0_ = -6715.60 | 37 vs. 38 | 1 | **307.48** | 43 A  350 T  361 T | 1.197 *±* 0.473  1.919 *±* 1.056  1.157 *±* 0.587 | 0.596  0.988*  0.553 |
|  |  | *ℓ*_1_ = -6561.86 |  |  |  |  |  |  |
|  | M1a vs. M2a (nearly neutral vs. positive selection) | *ℓ*_0_ = -6561.86 | 38 vs. 40 | 2 | 0.00 |  |  |  |
|  |  | *ℓ*_1_ = -6561.86 |  |  |  |  |  |  |
|  | M7 vs. M8 (beta vs. beta&ω) | *ℓ*_0_ = -6505.59 | 38 vs. 40 | 2 | **18.87** |  |  |  |
|  |  | *ℓ*_1_ = -6496.15 |  |  |  |  |  |  |
| *nad*1 | M0 vs. M1a (one-ratio vs. nearly neutral) | *ℓ*_0_ = -6303.78 | 37 vs. 38 | 1 | **201.08** | —— | —— | —— |
|  |  | *ℓ*_1_ = -6203.24 |  |  |  |  |  |  |
|  | M1a vs. M2a (nearly neutral vs. positive selection) | *ℓ*_0_ = -6203.24 | 38 vs. 40 | 2 | 0.00 |  |  |  |
|  |  | *ℓ*_1_ = -6203.24 |  |  |  |  |  |  |
|  | M7 vs. M8 (beta vs. beta&ω) | *ℓ*_0_ = -6120.61 | 38 vs. 40 | 2 | 3.53 |  |  |  |
|  |  | *ℓ*_1_ = -6118.85 |  |  |  |  |  |  |
| *nad*2 | M0 vs. M1a (one-ratio vs. nearly neutral) | *ℓ*_0_ = -1367.24 | 37 vs. 38 | 1 | **37.58** | —— | —— | —— |
|  |  | *ℓ*_1_ = -1348.45 |  |  |  |  |  |  |
|  | M1a vs. M2a (nearly neutral vs. positive selection) | *ℓ*_0_ = -1348.45 | 38 vs. 40 | 2 | 0.00 |  |  |  |
|  |  | *ℓ*_1_ = -1348.45 |  |  |  |  |  |  |
|  | M7 vs. M8 (beta vs. beta&ω) | *ℓ*_0_ = -1339.51 | 38 vs. 40 | 2 | 0.00 |  |  |  |
|  |  | *ℓ*_1_ = -1339.51 |  |  |  |  |  |  |
| *nad*3 | M0 vs. M1a (one-ratio vs. nearly neutral) | *ℓ*_0_ = -2664.05 | 37 vs. 38 | 1 | **50.58** | —— | —— | —— |
|  |  | *ℓ*_1_ = -2638.76 |  |  |  |  |  |  |
|  | M1a vs. M2a (nearly neutral vs. positive selection) | *ℓ*_0_ = -2638.76 | 38 vs. 40 | 2 | 0.00 |  |  |  |
|  |  | *ℓ*_1_ = -2638.76 |  |  |  |  |  |  |
|  | M7 vs. M8 (beta vs. beta&ω) | *ℓ*_0_ = -2598.68 | 38 vs. 40 | 2 | 1.23 |  |  |  |
|  |  | *ℓ*_1_ = -2598.06 |  |  |  |  |  |  |
| *nad*4 | M0 vs. M1a (one-ratio vs. nearly neutral) | *ℓ*_0_ = -655.88 | 37 vs. 38 | 1 | **11.50** | —— | —— | —— |
|  |  | *ℓ*_1_ = -650.13 |  |  |  |  |  |  |
|  | M1a vs. M2a (nearly neutral vs. positive selection) | *ℓ*_0_ = -650.13 | 38 vs. 40 | 2 | 0.00 |  |  |  |
|  |  | *ℓ*_1_ = -650.13 |  |  |  |  |  |  |
|  | M7 vs. M8 (beta vs. beta&ω) | *ℓ*_0_ = -649.92 | 38 vs. 40 | 2 | 0.00 |  |  |  |
|  |  | *ℓ*_1_ = -649.92 |  |  |  |  |  |  |
| *nad*4L | M0 vs. M1a (one-ratio vs. nearly neutral) | *ℓ*_0_ = -8610.66 | 37 vs. 38 | 1 | **134.61** | 254 L | 1.264 *±* 0.571 | 0.673 |
|  |  | *ℓ*_1_ = -8543.36 |  |  |  |  |  |  |
|  | M1a vs. M2a (nearly neutral vs. positive selection) | *ℓ*_0_ = -8543.36 | 38 vs. 40 | 2 | 0.00 |  |  |  |
|  |  | *ℓ*_1_ = -8543.36 |  |  |  |  |  |  |
|  | M7 vs. M8 (beta vs. beta&ω) | *ℓ*_0_ = -8449.38 | 38 vs. 40 | 2 | 0.20 |  |  |  |
|  |  | *ℓ*_1_ = -8449.28 |  |  |  |  |  |  |
| *nad*5 | M0 vs. M1a (one-ratio vs. nearly neutral) | *ℓ*_0_ = -6583.78 | 37 vs. 38 | 1 | **295.12** | 10 F  13 T  19 L  23 N  276 A  283 L | 1.145 *±* 0.455  1.058 *±* 0.538  1.112 *±* 0.477  1.265 *±* 0.382  1.514 *±* 0.405  1.264 *±* 0.454 | 0.574  0.501  0.554  0.693  0.948  0.695 |
|  |  | *ℓ*_1_ = -6436.22 |  |  |  |  |  |  |
|  | M1a vs. M2a (nearly neutral vs. positive selection) | *ℓ*_0_ = -6436.22 | 38 vs. 40 | 2 | 0.00 |  |  |  |
|  |  | *ℓ*_1_ = -6436.22 |  |  |  |  |  |  |
|  | M7 vs. M8 (beta vs. beta&ω) | *ℓ*_0_ = -6353.67 | 38 vs. 40 | 2 | **20.25** |  |  |  |
|  |  | *ℓ*_1_ = -6343.54 |  |  |  |  |  |  |
| *nad*6 | M0 vs. M1a (one-ratio vs. nearly neutral) | *ℓ*_0_ = -2904.93 | 37 vs. 38 | 1 | **93.51** | —— | —— | —— |
|  |  | *ℓ*_1_ = -2858.17 |  |  |  |  |  |  |
|  | M1a vs. M2a (nearly neutral vs. positive selection) | *ℓ*_0_ = -2858.17 | 38 vs. 40 | 2 | 0.00 |  |  |  |
|  |  | *ℓ*_1_ = -2858.17 |  |  |  |  |  |  |
|  | M7 vs. M8 (beta vs. beta&ω) | *ℓ*_0_ = -2828.21 | 38 vs. 40 | 2 | 0.00 |  |  |  |
|  |  | *ℓ*_1_ = -2828.21 |  |  |  |  |  |  |

Note: a. *ℓ*_0_ refers to the log-likelihood score for the null model, whereas *ℓ*_1_ represents the log-likelihood score under the alternative model. b. Free parameters include branch lengths (17 taxa tips ×2 – 3 = 31), the equilibrium frequencies (3), the transition/transversion rate ratio κ (1), and the parameters for the omega distribution according to Álvarez-Carretero et al.. c. The bold font indicates the significant difference between the compared two models. The critical values are χ2 _1, 5%_ = 3.84, and χ2 _2, 5%_ = 5.99, where 1 and 2 represent df and 5% indicates the significance level. d. The numbers in the positive selected codons column indicate the position of the codon, and the capital letter represent the codon abbreviation. * indicate codons identified as positively selected with posterior probability ＞ 0.95 estimated by Bayes empirical Bayes (BEB) analysis.

Table S5. Non-parametric likelihood-based tests of 5 alternative tree topologies recovered from this study. Five datasets correspond to the definition provided in Figure S3. Four topologies are defined as follows: T1 is the topology derived from *D*_PRT_, *D*_PT_; T2 is the topology derived from *D*_PR_; T3 is the topology derived from *D*_P_ using BI algorithms; T4 is the topology derived from *D*_P_ using ML algorithms; T5 is the topology derived from *D*_RT_. All tests are carried out by CONSEL. Bolded values indicate that the corresponding topologies are ruled out for the dataset at *p* < 0.05 level.

| Datasets | T1 | | | T2 | | | T3 | | | T4 | | | T5 | | |
| --- | --- | --- | --- | --- | --- | --- | --- | --- | --- | --- | --- | --- | --- | --- | --- |
|  | AU | KH | SH | AU | KH | SH | AU | KH | SH | AU | KH | SH | AU | KH | SH |
| *D*_PRT_ | 0.606 | 0.589 | 0.899 | 0.402 | 0.411 | 0.782 | **2.00E-06** | **2.00E-04** | **4.00E-04** | **1.00E-04** | **0.001** | **0.001** | **7.00E-62** | **0** | **0.016** |
| *D*_PR_ | 0.326 | 0.312 | 0.723 | 0.705 | 0.688 | 0.917 | **0.01** | **0.001** | **0.002** | **0.002** | **0.002** | **0.009** | **3.00E-07** | **0** | **0.011** |
| *D*_PT_ | 0.777 | 0.688 | 0.937 | 0.422 | 0.312 | 0.716 | 0.130 | 0.098 | 0.146 | 0.159 | 0.118 | 0.163 | **3.00E-41** | **0** | **0.024** |
| *D*_P_ | 0.479 | 0.393 | 0.796 | 0.651 | 0.607 | 0.884 | 0.259 | 0.264 | 0.375 | 0.410 | 0.317 | 0.485 | **1.00E-70** | **3.00E-05** | **0.015** |
| *D*_RT_ | 0.471 | 0.363 | 0.780 | 0.196 | 0.223 | 0.507 | **9.00E-06** | **0** | **0** | **4.00E-05** | **2.00E-04** | **2.00E-04** | 0.694 | 0.637 | 0.841 |

Note: AU, approximately unbiased test; KH, Kishino-Hasegawa test; SH, Shimodaira-Hasegawa test.

Table S6. Sequence partition scheme and nucleotide substitution models for Bayesian inference (BI) and maximum likelihood (ML) phylogenetic analyses for the best topology T1 of dataset *D*_PRT_.

| Sequence partition name | Partition schemes | Nucleotide substitution model |
| --- | --- | --- |
| Subset1 | *atp*6, *atp*8, *nad*1, *nad*2, *nad*3, *nad*4L, *nad*4, *nad*5, *cytb* | GTR+I+G |
| Subset2 | *cox*1, *cox*2, *cox*3 | GTR+I+G |
| Subset3 | *nad*6 | GTR+I+G |
| Subset4 | 12S, 16S | GTR + G |
| Subset5 | tRNA | GTR+I+G |

Table S7. Sequence partition scheme and nucleotide substitution priors for divergence time estimation. Six-digit numbers represent transition/transversion split models; α is the gamma shape, and Invar is the proportion invariant.

| Sequence partition name | Partition schemes | Nucleotide substitution priors |
| --- | --- | --- |
| Subset1 | *atp*6, *atp*8, *nad*1, *nad*2, *nad*3, *nad*4L, *nad*4, *nad*5 | 121345: TN93  α: 0.817  Invar: 0.334 |
| Subset2 | *cox*1, *cox*2, *cox*3, *cytb* | 121343: TN93  α: 0.967  Invar: 0.517 |
| Subset3 | *nad*6 | 121134: TN93  α: 0.619  Invar: 0.116 |
| Subset4 | 12S, 16S, *trn*D, *trn*F, *trn*G, *trn*H, *trn*I, *trn*K, *trn*L1, *trn*L2, *trn*M, *trn*R, *trn*S1, *trn*T, *trn*V, *trn*W | 123454: TVM  α: 0.379  Invar: 0.221 |
| Subset5 | *trn*A, *trn*C, *trn*E, *trn*N, *trn*P, *trn*Q, *trn*S2, *trn*Y | 123143: TN93  α: 0.316  Invar: 0.182 |


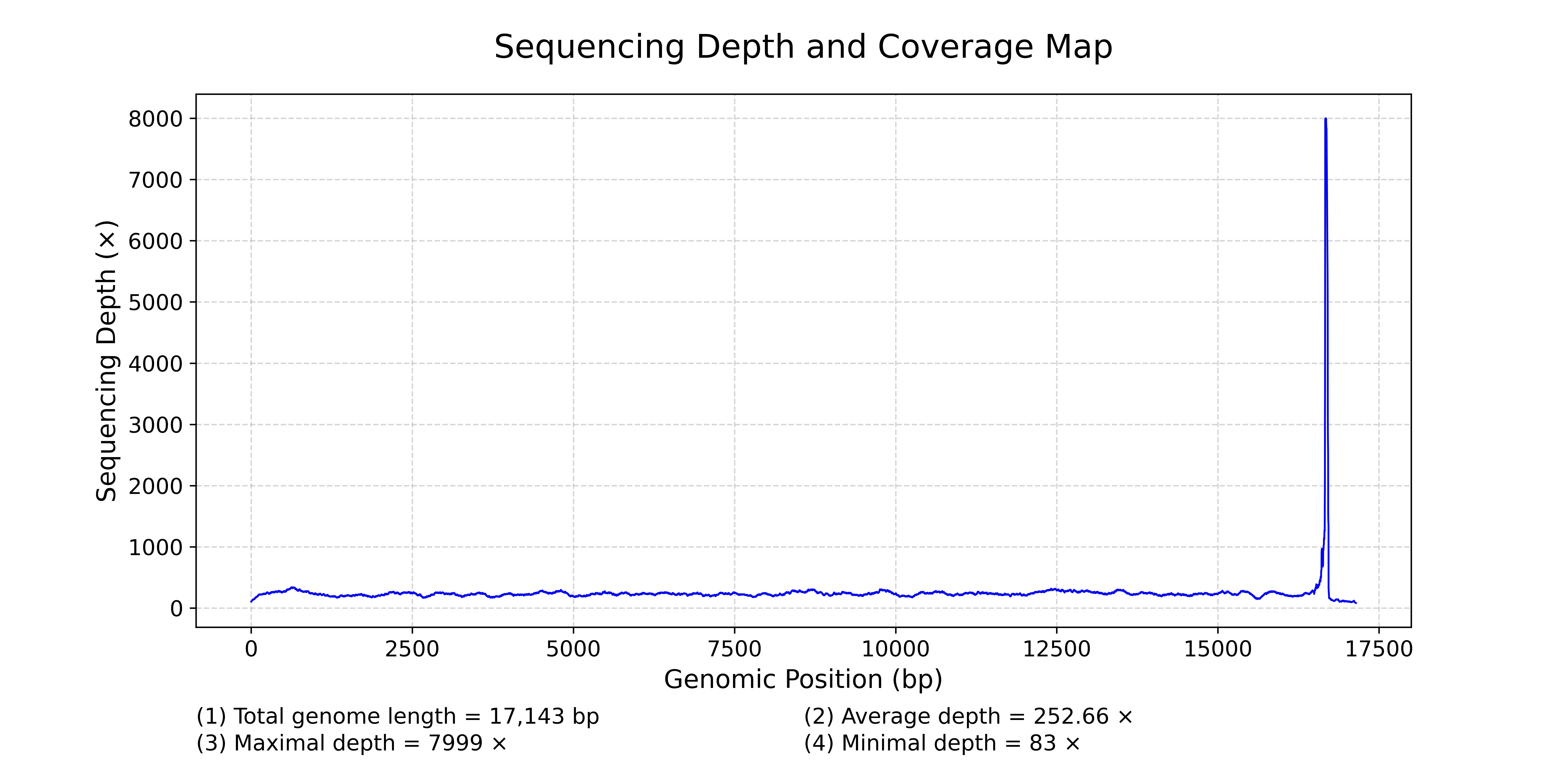


Figure S1. Sequencing depth and coverage map of *Pelodiscus axenaria* (OR805132) mitochondrial genome. The maximum sequencing depth reached 7999×, while the minimum was 83×, with an average depth of 252.66×. The map was created using Python script authored by Liu C, available at dx.doi.org/10.17504/protocols.io.4r3l27jkxg1y/v1.


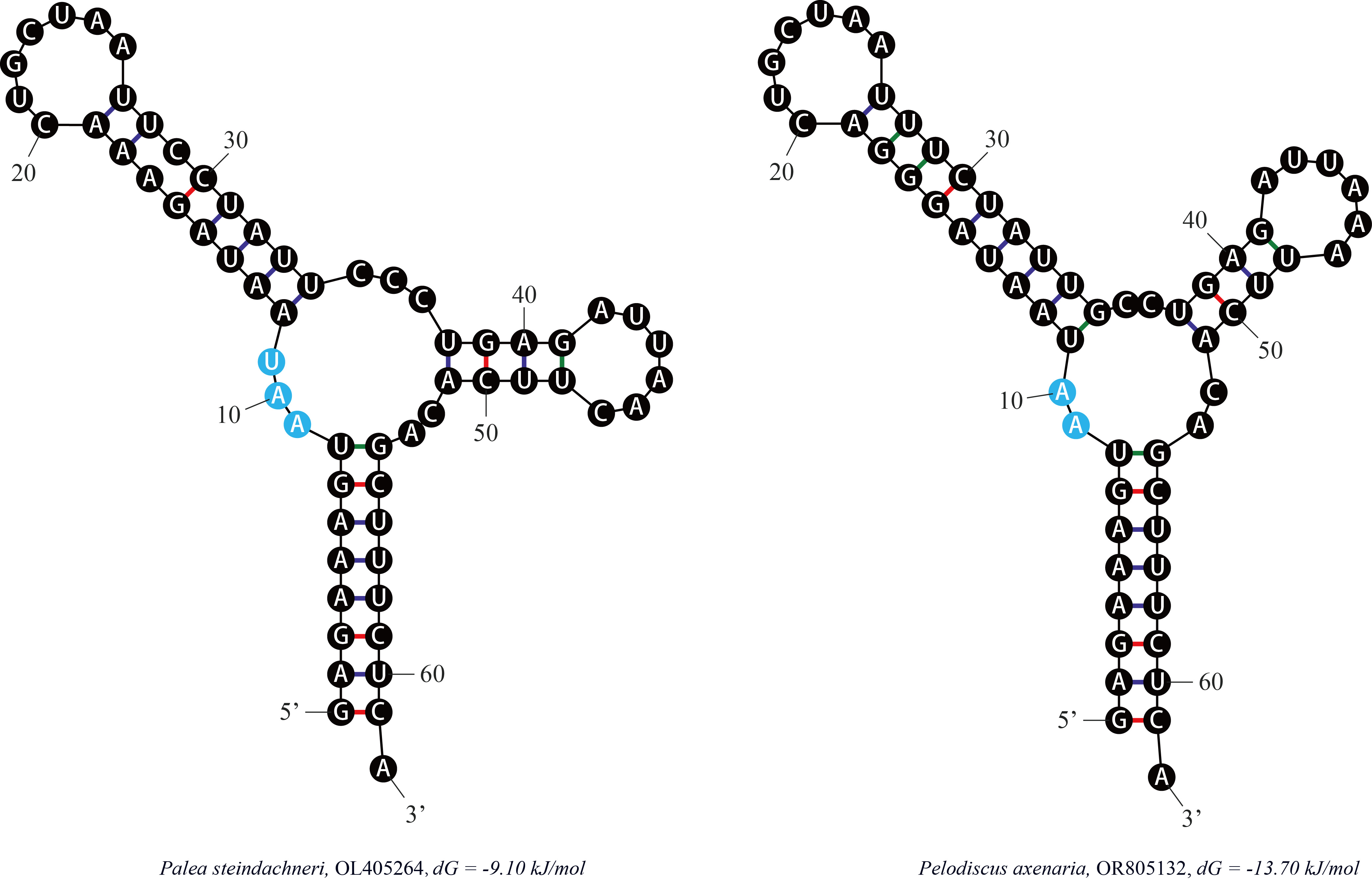


Figure S2. Potential secondary structures of tRNA^Ser (GCU)^ of the *Palea steindachneri* (OL405264, left) and *Pelodiscus axenaria* (OR805132, right) mitochondrial genomes. Inferred Watson-Crick bonds are illustrated by thin bars and wobble base pairs are illustrated by thick color bars. The bases symbols with the blue background represent the positions of the absent DHU-arm.

Figure S3. The amino acid alignment of the Trionychia *cytb* gene. The red arrows indicate the positive selection codon sites detected by the Bayes empirical Bayes (BEB) analysis, with only 350 T being significant.

_

_

Figure S4. Topologies of Trionychia inferred from five empirical datasets with *Mauremys reevesii* (FJ469674.1) and *Mauremys mutica* (NC 009330.1) as outgroups. The datasets are defined as follows: *D*_PRT_ = PCGs + rRNAs + tRNAs, *D*_PR_ = PCGs + rRNAs, *D*_PT_ = PCGs + tRNAs, *D*_RT_ = rRNAs + tRNAs, and *D*_P_ = PCGs. For each dataset, the Bayesian trees are placed on the left and the maximum likelihood trees are on the right. Numbers adjacent to the nodes are posterior probabilities and bootstraps, respectively. Scale bar represents the substitution rate per site. The internal nodal supports vary widely among datasets, and the *D*_PRT_ trees have the most strongly supported internal relationships with Bayesian posterior probabilities (PPs) of 1.00 and bootstrap proportions (BPs) of higher than 76%.

**References**

Amer, S. and Kumazawa, Y. Complete sequence of the mitochondrial genome of the endangered Nile soft-shelled turtle *Trionyx triunguis*. The Egyptian Journal of Experimental Biology (Zoology), 2009, **5**: 43-50.

Álvarez-Carretero S, Kapli P, and Yang Z Beginner's Guide on the use of PAML to detect positive selection [J]. Molecular Biology and Evolution, 2023, **40**: 1-18.

Baek, H. J., Kim, P., Kim, Y. C., et al. The complete mitochondrial genome of the Amur soft-shelled turtle (*Pelodiscus maackii* Brandt, 1858), from South Korea. Mitochondrial DNA Part B: Resources, 2022, **7**: 498-500.

Chen, C., Hong, X. Y., Li, W., et al. Complete mitochondrial genome and the phylogenetic position of the Burmese narrow-headed softshell turtle *Chitra vandijki* (Testudines: Trionychidae). Mitochondrial DNA Part B, 2021, **6**: 1216-1218.

Chen, X., Zhou, Z. M., Peng, X., et al. Complete mitochondrial genome of the endangered Asian giant softshell turtle *Pelochelys cantorii* (Testudinata: Trionychidae). Mitochondrial DNA, 2013, **24**: 111-113.

Cui, L., Rao, D. Q., and Zhang, M. W. The complete mitochondrial genome of *Amyda cartilaginea* (Testudines: Trionychidae). Mitochondrial DNA Part B: Resources, 2020, **5**: 3670-3672.

Fritz, U., Gong, S. P., Auer, M., et al. The world’s economically most important chelonians represent a diverse species complex (Testudines: Trionychidae: *Pelodiscus*). Organisms Diversity & Evolution, 2010, **10**: 227-242.

Jung, S. O., Lee, Y. M., Kartavtsev, Y., et al. The complete mitochondrial genome of the Korean soft-shelled turtle *Pelodiscus sinensis* (Testudines, Trionychidae). DNA Sequence - Journal of DNA Sequencing and Mapping, 2006, **17**: 471-483.

Kundu, S., Kumar, V., Laskar, B. A., et al. Complete mitochondrial genome of black soft-shell turtle (*Nilssonia nigricans*) and comparative analysis with other Trionychidae. Scientific Reports, 2018, **8**: 1-11.

Li, H. F., Liu, J. J., Xiong, L., et al. Phylogenetic relationships and divergence dates of softshell turtles (Testudines: Trionychidae) inferred from complete mitochondrial genomes. Journal of Evolutionary Biology, 2017, **30**: 1011-1023.

Li, X. S., Nie, L. W., Wang, L., et al. The mitochondrial genome complete sequence and organization of the Pig-nosed turtle *Carettochelys insculpta* (Testudines, Carettochelyidae) and its phylogeny position in Testudines. Amphibia Reptilia, 2010, **31**: 541-551.

Yu, P., Yang, X. L., Zhou, W. S., et al. Comparative mitogenomic and phylogenetic analysis of *Apalone spinifera* and *Apalone ferox* (Testudines: Trionychidae). Genetica, 2019, **147**: 165-176.

Zhang, J., Zhou, Q. Y., Yang, X. L., et al. Characterization of the complete mitochondrial genome and phylogenetic analysis of *Pelodiscus sinensis*, a mutant Chinese soft-shell turtle. Conservation Genetics Resources, 2019, **11**: 279-282.

Zhang, X. C., Li, W., Zhao, J., et al. The complete mitogenome of *Pelochelys cantorii* (Guangning) and the comparative analysis of different habitats. Conservation Genetics Resources, 2018, **10**: 17-20.
