# Supplementary material for: Comparative analysis of the mitochondrial genomes of the soft-shelled turtles Palea steindachneri and Pelodiscus axenaria and phylogenetic implications for Trionychia
Source: Sci Rep. 2025 Feb 28;15:7138. doi: 10.1038/s41598-025-90985-2 (PMC11871352; doi:10.1038/s41598-025-90985-2)
Supplement: Supplementary file 3 — Supplementary Material 3 [file 41598_2025_90985_MOESM3_ESM.pdf]

Appendices I, II and III

valid from 7 February 2025

Interpretation

1. Species included in these Appendices are referred to:

   a) by the name of the species; or
   b) as being all of the species included in a higher taxon or designated part thereof.

2. The abbreviation “spp.” is used to denote all species of a higher taxon.

3. Other references to taxa higher than species are for the purposes of information or classification only. The common names included after the scientific names of families are for reference only. They are intended to indicate the species within the family concerned that are included in the Appendices. In most cases this is not all of the species within the family.

4. The following abbreviations are used for plant taxa below the level of species:

   a) “ssp.” is used to denote subspecies; and
   b) “var(s).” is used to denote variety (varieties).

5. As none of the species or higher taxa of FLORA included in Appendix I is annotated to the effect that its hybrids shall be treated in accordance with the provisions of Article III of the Convention, this means that artificially propagated hybrids produced from one or more of these species or taxa may be traded with a certificate of artificial propagation, and that seeds and pollen (including pollinia), cut flowers, seedling or tissue cultures obtained *in vitro* transported in sterile containers of these hybrids are not subject to the provisions of the Convention.

6. The names of the countries in parentheses placed against the names of species in Appendix III are those of the Parties submitting these species for inclusion in this Appendix.

7. When a species is included in Appendix I, II or III, the whole, live or dead, animal or plant is always included. In addition, all parts and derivatives thereof are also included in the same Appendix unless, for animal species listed in Appendix III and plant species listed in Appendix II or III, the species is annotated with the symbol # followed by a number to indicate that only specific parts and derivatives are included. The symbol # followed by a number placed against the name of a species or higher taxon included in Appendix II or III refers to a footnote that indicates the parts or derivatives of animals or plants that are designated as 'specimens' subject to the provisions of the Convention in accordance with Article I, paragraph (b), subparagraph (ii) or (iii).

8. The terms and expressions below, used in annotations in these Appendices, are defined as follows:

   Extract

   Any substance obtained directly from plant material by physical or chemical means regardless of the manufacturing process. An extract may be solid (e.g. crystals, resin, fine or coarse particles), semi-solid (e.g. gums, waxes) or liquid (e.g. solutions, tinctures, oil and essential oils).

   Finished musical instruments

   A musical instrument (as referenced by the Harmonized System of the World Customs Organization, Chapter 92; musical instruments, parts and accessories of such articles) that is ready to play or needs only the installation of parts to make it playable. This term includes antique instruments (as defined by the Harmonized System codes 97.05 and 97.06; Works of art, collectors' pieces and antiques).

Finished musical instrument accessories

A musical instrument accessory (as referenced by the Harmonized System of the World Customs Organization, Chapter 92; musical instruments, parts and accessories of such articles) that is separate from the musical instrument, and is specifically designed or shaped to be used explicitly in association with an instrument, and that requires no further modification to be used.

Finished musical instrument parts

A part (as referenced by the Harmonized System of the World Customs Organization, Chapter 92; musical instruments, parts and accessories of such articles) of a musical instrument that is ready to install and is specifically designed and shaped to be used ex[1]plicitly in association with the instrument to make it playable.

Finished products packaged and ready for retail trade

Products, shipped singly or in bulk, requiring no further processing, packaged, labelled for final use or the retail trade in a state fit for being sold to or used by the general public.

Powder

A dry, solid substance in the form of fine or coarse particles.

Shipment

Cargo transported under the terms of a single bill of lading or air waybill, irrespective of the quantity or number of containers or packages; or pieces worn, carried or included in personal baggage.

Ten (10) kg per shipment

For the term "10 kg per shipment", the 10 kg limit should be interpreted as referring to the weight of wood of each individual annotated species of genus Dalbergia *or* Guibourtia *present in the items in the shipment. The 10 kg limit is to be assessed only against the individual weights of the portions of wood of each individual annotated species contained in each item of the shipment, and not against the total weight of the shipment. The total weights present of each individual annotated species are considered individually to determine whether a CITES permit or certificate is required for each individual annotated species, and weights of different individual annotated species are not added together for this purpose.*

Transformed wood

Defined by Harmonized System code 44.09: Wood (including strips, friezes for parquet flooring, not assembled), continuously shaped (tongued, grooved, rebated, chamfered, V-jointed, beaded, moulded, rounded or the like) along any of its edges, ends or faces, whether or not planed, sanded or end-jointed.

Woodchips

Wood that has been reduced to small pieces.

1

	Appendices I	II	III
	Apalone spinifera atra		
		***Chitra* spp.** (Except the species included in Appendix I)	
	Chitra chitra		
	Chitra vandijki		
		Cyclanorbis elegans	
		Cyclanorbis senegalensis	
		Cycloderma aubryi	
		Cycloderma frenatum	
		Dogania subplana	
		Lissemys ceylonensis	
		Lissemys punctata	
		Lissemys scutata	
		Nilssonia formosa	
	Nilssonia gangetica		
	Nilssonia hurum		
	Nilssonia leithii		
	Nilssonia nigricans		
		Palea steindachneri	
		***Pelochelys* spp.**	
		Pelodiscus axenaria	
		Pelodiscus maackii	
		Pelodiscus parviformis	
		Rafetus euphraticus	
		Rafetus swinhoei	
		Trionyx triunguis	
